# Transient directing group enabled Pd-catalyzed C–H oxygenation of benzaldehydes and benzylic amines†

**DOI:** 10.1039/d2ra00241h

**Published:** 2022-06-27

**Authors:** Mixiang Tian, Lidong Shao, Xiaosan Su, Xuhong Zhou, Honglei Zhang, Kun Wei, Ruifen Sun, Junliang Wang

**Affiliations:** Center for Scientific Research, Yunnan University of Chinese Medicine Kunming Yunnan 650500 P. R. China wangjunliangedu@163.com sunruifen@ynutcm.edu.cn; School of Chemical Science and Technology, Yunnan University Kunming Yunnan 650500 P. R. China

## Abstract

We report a general protocol for <i>ortho</i>-C–H fluoroalkoxylation of benzaldehydes and benzylic amines utilizing an inexpensive amino amide as a transient directing group. In the presence of an electrophilic fluorinating bystanding oxidant and fluorinated alcohols, a wide range of benzaldehydes and benzylic amines could be oxygenated selectively at the ortho positions to afford fluoroalkyl aryl ethers. This elegant approach would provide appealing strategies for synthesis of drug molecules and natural products.

## Abstract 2

A general protocol for ortho-C–H fluoroalkoxylation of benzaldehydes and benzylic amines was exploited by utilizing an inexpensive amino amide as a transient directing group.

## Graphical abstract

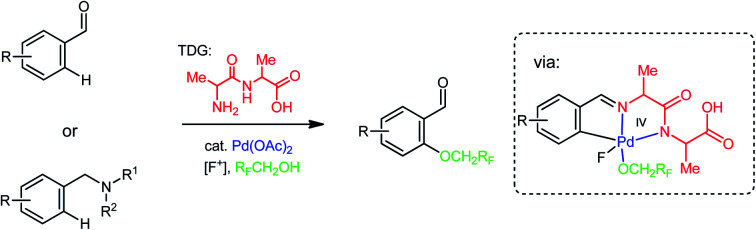

## Introduction

The directed activation of carbon–hydrogen bonds (C–H) is a topic of increasing importance with a wide range of applications in organic synthesis.^1^ However, one of the major challenges in selective, catalytic functionalization of C–H bonds is to enable the use of native substrates without covalently installing external directing groups. Transient directing groups (TDGs), which form reversible linkages with the substrates *in situ*, complement a promising alternative approach to direct metal-catalysed or -mediated C–H functionalization, thereby avoiding the additional installation and removal of directing groups.^2^ While most of the achievements for TDG-mediated and Pd-catalyzed C(sp^2^)–H functionalization are restricted in C(sp^2^)–H arylation,^3^ alkylation,^4^ and alkenylation,^5^ efforts to develop Pd-catalyzed C(sp^2^)–O and C(sp^2^)–N bond forming reactions have recently afforded new advances.^6^ Recently, the seminal examples of these rare reactions, which were reported by Erik J. Sorensen and co-workers, featured the utilization of 4-chloroanthranilic acid as the bidentate transient directing group (BiTDG), and 1-fluoro-2,4,6-trimethylpyridnium triflate as the bystanding oxidant.^7^ Soon after, *ortho*-C–H methoxylation and fluoroalkoxylation of benzaldehydes were also realized by employing monodentate directing groups^8*a*^ and BiTDG respectively which form *N*, *O*-bis-coordinated complexes with Pd(ii) catalyst and promote the C–H activation process.^8*b*^ Nevertheless, mild and selective transformations of this type are still largely undeveloped and the new catalytic TDGs, *e.g.*, *N*, *N*-bidentate coordination, remain to be further exploited. Therefore, the investigation of new TDGs allowing flexible and diverse reactivities is of great importance and highly desirable.

**Scheme 1:**
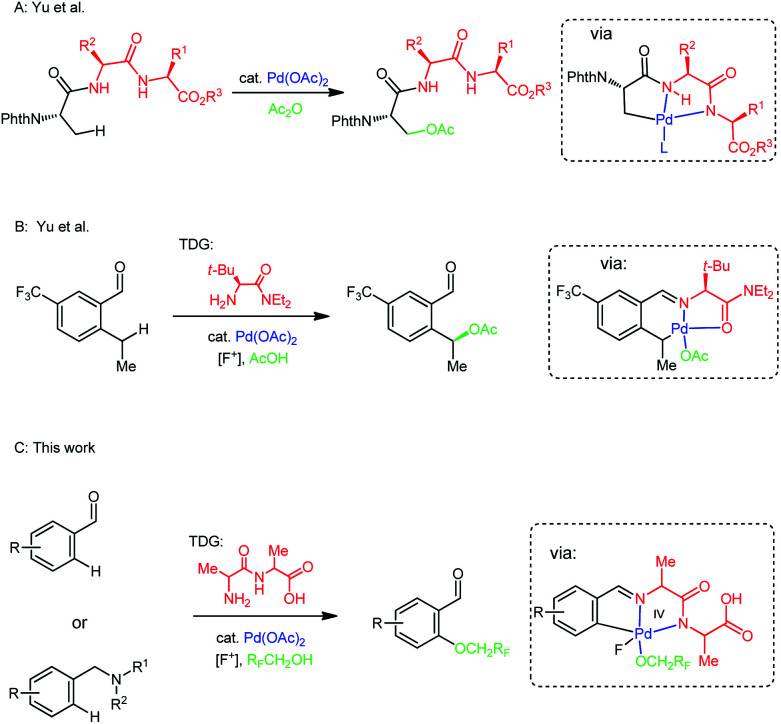
Controlled site-selective C–H bond oxygenation.

The use of the peptide backbone in site-selective C–H oxygenations of tripeptides presented catalytic action by *N*, *N*-bidentate coordination of amide groups with Pd (Scheme 1A).^9^ Significantly, a bulky, amino amide transient directing group was developed to selectively promote the benzylic C(sp^3^)–H acetoxylation with low yield (29%) (Scheme 1B).^10^ Based on this particular investigation and our previous studies of using amino amide as catalytic transient directing group enabled C(sp^2^)–H activation *via N*, *N*-bidentate coordination with Pd catalyst,^3*d*,*k*^ we speculated that an amino amide could serve as a suitable TDG which could form a *N*, *N*-coordinated complex with Pd(ii) to enable subsequent C(sp^2^)–H oxidation in a manner similar to the *N*,*N*-bidentate coordination in the tripeptides, and decided to explore the feasibility of oxidation of benzaldehydes using amino amide transient directing group. Herein, we report an amino amide as TDG utilized to promote C(sp^2^)–H oxygenation of broad range of benzaldehydes and benzylic amines using *N*-fluoro-2,4,6-trimethylpyridinium tetrafluoroborate as the bystanding oxidant (Scheme 1C), the protocol features a one-step coupling between a wide range of benzaldehydes and benzylic amines and alcohols *via* C–O bond formation at the unactivated positions.^11^

To establish the viability of the C(sp^2^)–H oxygenation of benzaldehydes, diverse molecules containing amino groups were investigated their role in palladium-catalyzed oxygenation of benzaldehyde (1a) with trifluoroethanol (2a) in the presence of 10 mol% of Pd(OAc)_2_, 2 equiv. of TFA and 2 equiv. of K_2_S_2_O_8_ (Table 1). First, commercially available glycinamides, such as 2-aminoacetamide hydrochloride (A1) and 2-aminopropanamide hydrochloride (A2), and hydrazides, such as picolinohydrazide (A3) and acetohydrazide (A4), were employed as TDGs for C(sp^2^)–H oxygenation and all afforded low yield. To further explore the efficiency of TDG, the screening of selected short peptides was conducted. It turned out that the reaction failed with 2-(2-aminoacetamido)acetic acid (A5) as the TDG, inversely its analogue, 2-(2-(2-aminoacetamido)acetamido)- acetic acid (A6), exhibited moderate catalytic activity. In contrast, all substituted peptides (A7–A9) led to the formation of the desired oxidation products (3a) compared with A5, and A9 afforded the best result. Then, a control experiment without a TDG afforded no desired product, indicating that TDGs are crucial to this reaction.

**Table 1:** TDG evaluation for the C(sp^2^)–H oxygenation of benzaldehydes^a^

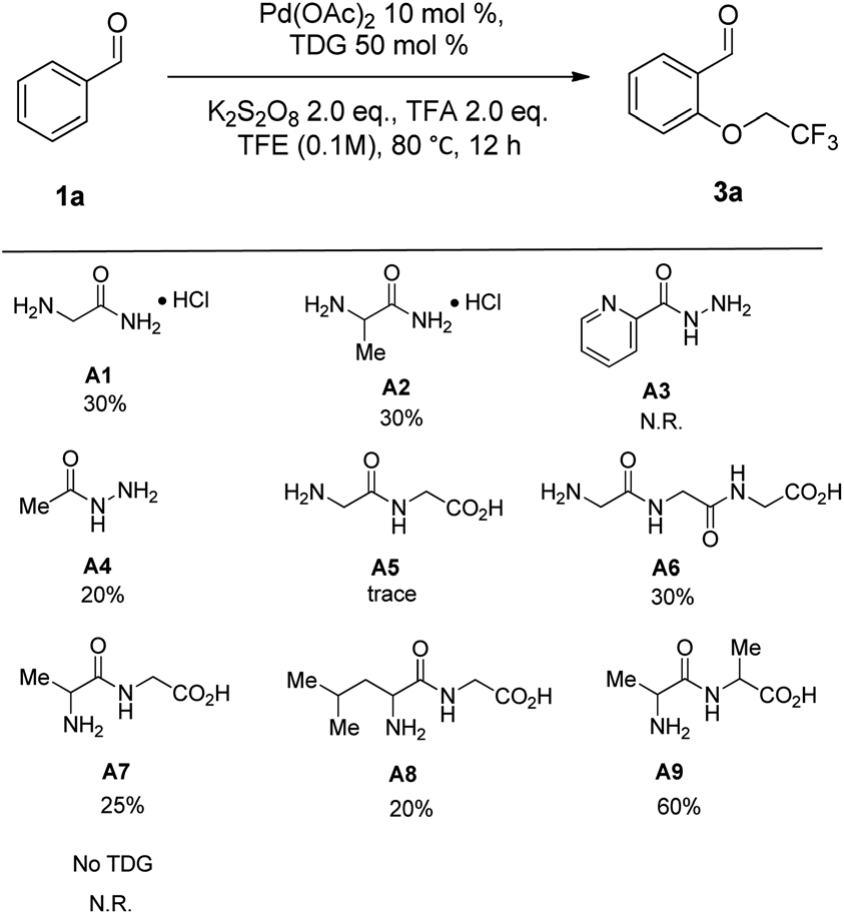

a Reaction conditions: 1a (0.2 mmol), TFE (2 mL), Pd(OAc)_2_ (10 mol%), TDG (50 mol%), K_2_S_2_O_8_ (0.4 mmol), TFA (2 equiv.) and stirred at 80 °C for 12 h. The per-centages under the chemical structures are their isolated yields.

With the best TDG in the presence of K_2_S_2_O_8_ identified, we tested different oxidants and found that 1-fluoro-2,4,6-trimethylpyridinium tetrafluoroborate F1 resulted in a better yield (Table 2, entries 1–5). Screening of the amount of TFA indicated that either low or high loading was not beneficial for the reaction (Table 2, entries 6–10), and 2 equiv. of TFA were still preferred. Other reaction parameters, including reaction temperature and solvents, were examined. Decreases in the reaction temperature led to an obvious drop in the yield (Table 2, entries 11 and 12). Compared with trifluoroethanol, other solvents, such as toluene and dichloromethane, were not applicable to the reaction (Table 2, entries 13–17). Additionally, 2-aminopropanoic acid was used as a contrast TDG under the optimum conditions, only 35% yield was achieved (Table 2, entry 18). Therefore, it can be presumed that the short peptides rather than its, hydrolysate can coordinate with Pd(ii) *via N*, *N*-bidentate coordination and further promote the C–H activation process. Interestingly, a lower yield was observed with either increased or decreased loading of the A9 (Table 2, entries 19 and 20). Lastly, control experiments show both Pd(OAc)_2_ and the oxidant F1 are essential for this transformation (Table 2, entry 21 and 22).

**Table 2:** Optimization of the reaction conditions^a^

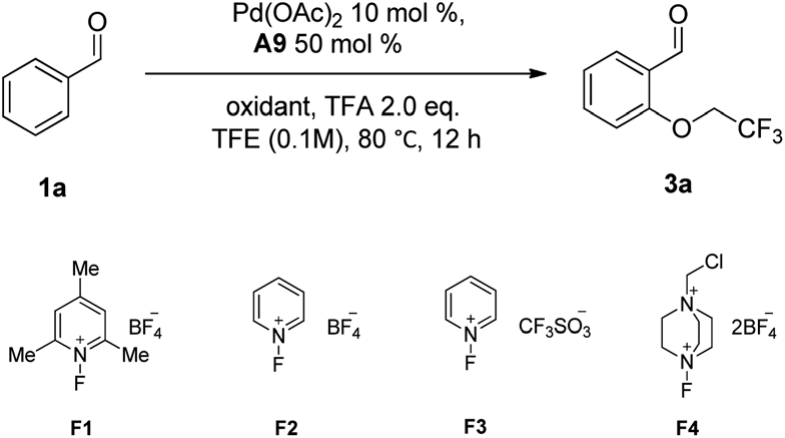			
Entry	Oxidant	TFA (equiv.)	Yield^b^(%)
1	F1	2	88
2	F2	2	60
3	F3	2	45
4	F4	2	60
5	Other oxidants^c^	2	<30
6	F1	0	30
7	F1	1	50
8	F1	3	80
9	F1	5	78
10	F1	10	78
11^d^	F1	2	75
12^e^	F1	2	60
13^f^	F1	2	Trace
14^g^	F1	2	30
15^h^	F1	2	Trace
16^i^	F1	2	Trace
17^j^	F1	2	25
18^k^	F1	2	35
19^l^	F1	2	30
20^m^	F1	2	72
21^n^	F1	2	N.R.
22	—	2	N.R.

a Reaction conditions: 1a (0.2 mmol), TFE (2 mL), [Pd] (10 mol%), TDG (50 mol%), oxidant (0.4 mmol), TFA (2 equiv.) and stirred at 80 °C for 12 h.

b Isolated yield.

c Other oxidants: (NH_4_)_2_S_2_O_8_, Na_2_S_2_O_8_, PhI(OAc)_2_, AgTFA, AgOAc, Ag_2_CO_3_, Ag_2_O.

d 60 °C.

e 40 °C.

f Solvent: TFE/Toluene (v/v, 1 : 1).

g Solvent: TFE/DCM (v/v, 1 : 1).

h DCM as solvent, 10 equiv. TFE.

i DCM as solvent, 20 equiv. TFE.

j DCM as solvent, 30 equiv. TFE.

k 2-Aminopropanoic acid instead of A9.

l Reaction performed with 0.3 equiv. of A9.

m Reaction performed with 0.7 equiv. of A9.

n without Pd(OAc)_2_.

**Scheme 2:**
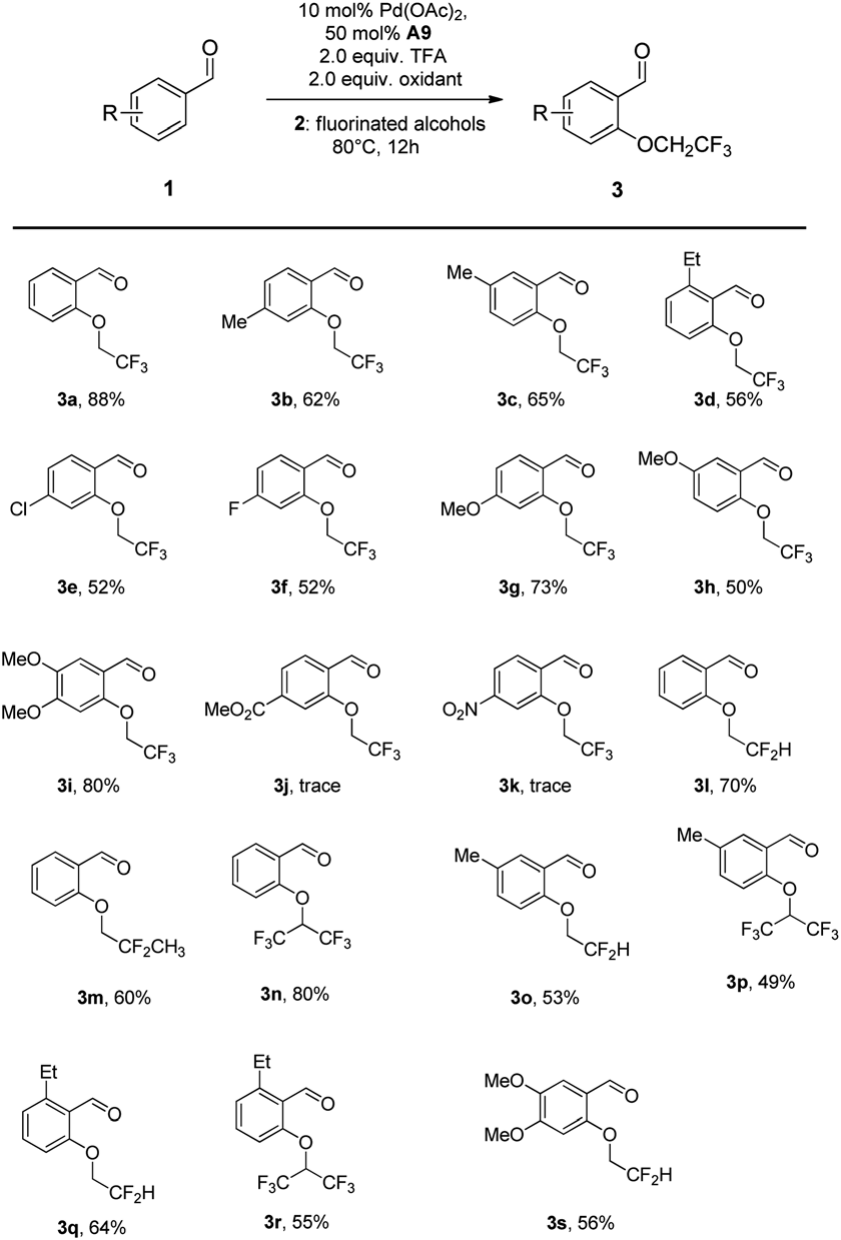
Scope of aldehydes and fluorinated alcohols.^*a a*^ Reaction conditions: 1 (0.2 mmol), fluorinated alcohols (2 mL), Pd(OAc)_2_ (10 mol%), A9 (50 mol%), F1 (0.4 mmol), TFA (2 equiv.) and stirred at 80 °C for 12 h. All yields given are those for the isolated products.

Under optimal conditions, we next investigated the substrate scope of substrates of benzaldehydes and fluorinated alcohols (Scheme 2). Firstly, the substrate scope of the various benzaldehydes was examined for the C(sp^2^)–H bond trifluoroethoxylation. We were delighted to find that different benzaldehydes bearing the alkyl group, halogen and the electron-rich methoxy group substituents were well tolerated to give the desired products in moderate to good yields (3a–3i).

When we changed the placement of the methyl or methoxyl group from the para-position to the meta-position, satisfactory results were also obtained (3b, 3c, 3g and 3h). It is noteworthy that 2-ethylbenzaldehyde also showed reaction activity in spite of the steric hindrance effect, affording the corresponding product in moderate yields (3d). Whereas, strong electron-deficient benzaldehydes with ester and nitro substituents only provided trace amounts of the product (3j and 3k). Furthermore, we also screened the other fluorinated alcohols under the standard conditions for obtaining fluorinated compounds. Gratifyingly, polyfluoroalkoxylation of benzaldehyde with other polyfluoro-alcohols proceeded smoothly with completely selectivity in moderate yields (3l–3n).^12^ Significantly, the *ortho*-C–H di-polyfluoroalkoxylation products could not be detected, demonstrating that the transformation showed good selectivity for the mono-fluoroalkoxylation. Moreover, 3-methylbenz-aldehyde and 3, 4-dimethoxy-benzaldehyde also afforded the corresponding mono-polyfluoroalkoxylated products in 49–64% yields (3o, 3p and 3s). Notably, secondary fluorinated alcohols such as 1,1,1,3,3,3-hexafluoro-2-propanol were also effective nucleophiles towards the benzaldehyde with steric hindrance, affording the highly hindered fluoroalkyl ethers in 55% isolated yields (3r).

Since the current reaction conditions were oxidative, we next wondered whether benzylic amines could be oxidized and serve as effective coupling partners for this reaction. Benzylamine instead of benzaldehyde was used as a substrate, and reacted with trifluoroethanol under the optimal reaction conditions (Table 3). To our delight, the C–N and ortho-C(sp^2^)–H consecutive oxidation of benzylamine could also be accomplished in one pot, albeit with lower efficiency (Table 3, entries 1). Upon evaluation of various oxidants, we discovered that although the [F+] bystanding oxidants gave inferior yields of the corresponding products in less than 10% (Table 3, entry 3–5), K_2_S_2_O_8_ was identified as potentially effective oxidant for this reaction, affording the desired product in 70% yield (Table 3, entry 2). Interestingly, the reaction with dual oxidant (F1/F4) system could be further optimized up to 80% isolated yield (Table 3, entry 6–10).

**Table 3:** Optimization of the consecutive oxidation of benzylamine

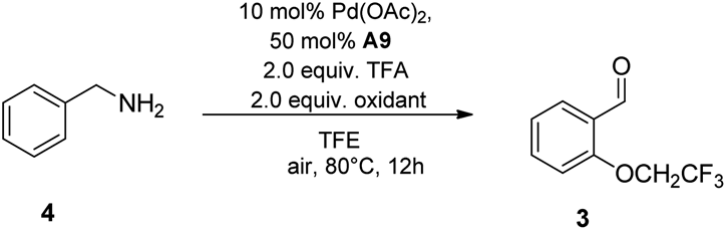			
Entry	Oxidant	Temperature(°C)	Yield^a^ (%)
1	F1	80	18%
2	K_2_S_2_O_8_	80	70%
3	F2	80	Trace
4	F3	80	Trace
5	F4	80	10%^b^ (86%)
6^c^	F1+ K_2_S_2_O_8_	80	78%
7^d^	F1+F4	80	80%
8^d^	F1+F4	60	70%
9^d^	F1+F4	50	55%
10^e^	F1+F4	80	78%

a Isolated yields.

b Benzaldehyde was formed as major product.

c F1 (0.2 mmol), K_2_S_2_O_8_ (0.2 mmol), under air.

d F1 (0.2 mmol), F4 (0.2 mmol), under air.

e F1 (0.2 mmol), F4 (0.2 mmol), under N_2_.

**Scheme 3:**
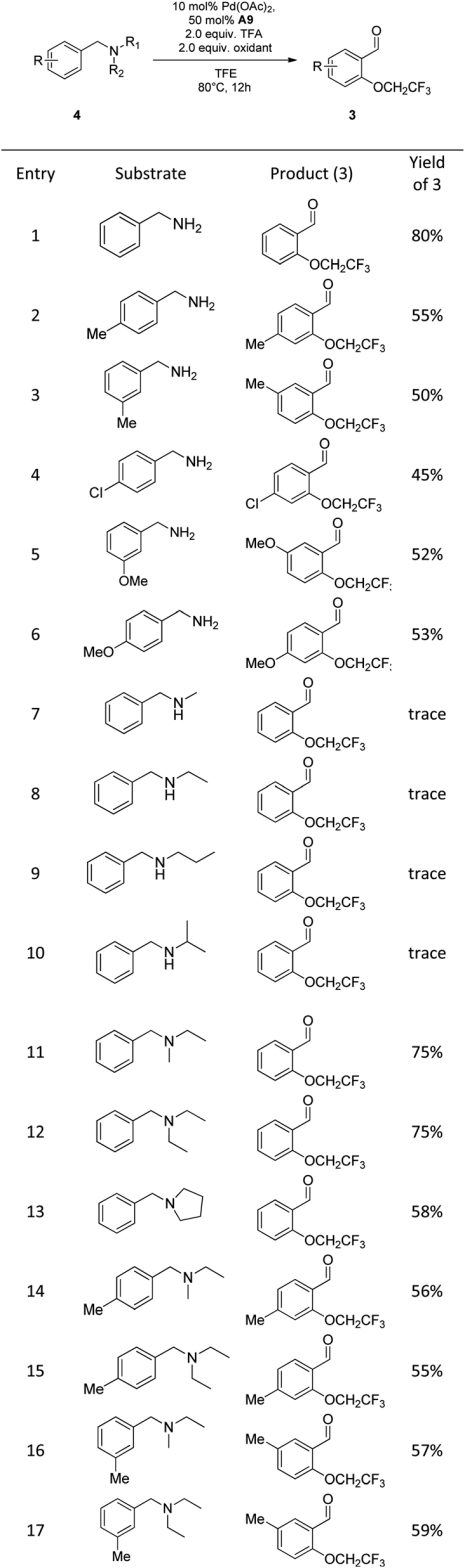
Scope of benzylic amines.^*a a*^ Reaction conditions: 4 (0.2 mmol), TFE (2 mL), Pd(OAc)_2_ (10 mol%), A9 (50 mol%), F1 (0.2 mmol), F4 (0.2 mmol), TFA (2 equiv.) and stirred at 80 °C for 12 h. All yields given are those for the isolated products.

On the basis of this strategy, a variety of amines were investigated under new conditions. *N*-benzyl primary amines having methyl group, halogens and the electron-rich methoxy group on the aromatic ring all underwent the desired oxidation reaction in 45–80% yields (Scheme 3, entry 1–6). Unfortunately, *N*-benzyl secondary amines, such as *N*-methyl-1-phenylmethanamine and *N*-benzylpropan-1-amine, only provided trace amounts of yield, which indicated that the secondary amines are not suitable for this process (Scheme 3, entry 7–10). Remarkably, *N*-benzyl tertiary amines were competent to undergo the reaction in moderate yields (Scheme 3, entry 11–17).

**Scheme 4:**
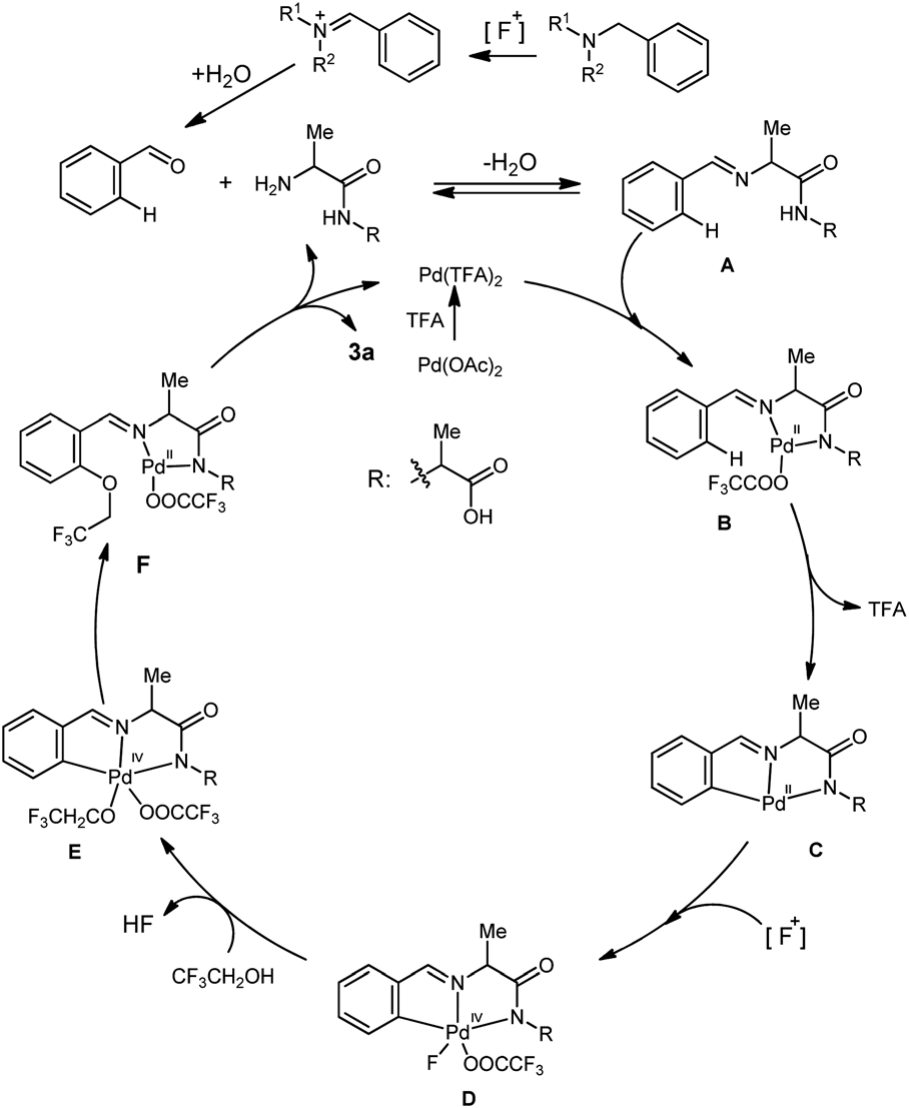
Proposed mechanism.

Based on our experiments and related literature,^3*d*,*k*,6–8^ a putative mechanism was proposed for the palladium-catalyzed *ortho*-C(sp^2^)–H oxidation of benzaldehydes (Scheme 4). Benzyl amines get converted into imine intermediate by the [F^+^] bystanding oxidant, which in turn could be hydrolyzed to the aldehydes. Condensation of benzaldehydes with the ligand 2-(2-aminopropanamido)propanoic acid provides the imine intermediate A. Coordination of this imine to a palladium species followed by cyclopalladation process generates the [5, 5]-bicyclic palladium intermediate C*via* a site-selective C–H bond activation process, and oxidative addition of the intermediate C with fluorinating bystanding oxidant generates the palladium(iv) species D. Next, the fluoride anion is displaced by the fluorinated alcohol to provide intermediate E. Reductive elimination of this palladium complex followed by a ligand dissociation process provides the α-imino amide, which releases the desired product, and ligand 2-(2-aminopropanamido)propanoic acid.

## Conclusions

In summary, we have developed a general method for the C(sp^2^)–H fluoroalkoxylation of benzaldehydes and benzylic amines using 2-(2-aminopropanamido)propanoic acid as the transient directing group and the [F^+^] bystanding oxidant. Moderate to good yields were obtained for a broad substrate scope under mild conditions. Given the efficient and selective processes for the functionalization of C(sp^2^)–H bonds, the approach will greatly enrich the toolbox and provide alternatively method for transformation of advanced synthetic intermediates in a single step. More importantly, the approach should find broad applications in synthesis of ubiquitous structural units in biologically active natural products and pharmaceuticals. Detailed mechanistic studies and new applications of this TDG strategy are underway in our laboratory.

## Experimental

### Typical procedure for palladium-catalyzed C(sp^2^)–H polyfluor-oalkoxylation of benzaldehydes

An 8 mL vial equipped with a stir bar was charged with Pd(OAc)_2_ (4.5 mg, 0.02 mmol, 10 mol%), A9 (16.0 mg, 0.1 mmol, 50 mol%), 1-fluoro-2,4,6-trimethylpyrdinium triflate (F1) (90.8 mg, 0.4 mmol, 2.0 equiv.), and benzaldehydes (0.2 mmol, 1.0 equiv.), followed by the addition of fluorinated alcohols (2.0 mL) and TFA (2.0 equiv.). The flask was then sealed and the mixture was stirred at room temperature for 10 min before being heated to 80 °C for 12 h. The reaction mixture was cooled to room temperature, followed by the addition of water and ethyl acetate. The organic layer was separated and the aqueous layer was extracted with ethyl acetate. The combined organic layer was washed with brine and dried over anhydrous MgSO_4_, filtrated and concentrated *in vacuo*, the residue was purified through column chromatography on silica gel to give the desired products.

### Typical procedure for the C–N and *ortho*-C(sp^2^)–H consecutive oxidation of benzylamines

Selectfluor (F4) (70.8 mg, 0.2 mmol, 1.0 equiv.) and 1-fluoro-2,4,6-trimethylpyrdinium triflate (F1) (90.8 mg, 0.2 mmol, 1.0 equiv.) were added to a solution of benzylamines (0.2 mmol, 1.0 equiv.) in fluorinated alcohols (2.0 mL). The reaction mixture was stirred at 80 °C for 30 min, followed by the addition of Pd(OAc)_2_ (4.5 mg, 0.02 mmol, 10 mol%), A9 (16.0 mg, 0.1 mmol, 50 mol%), and TFA (2.0 equiv.). The reaction mixture was stirred at the same temperature for 12 h. Upon completion, the reaction mixture was cooled to room temperature, followed by the addition of water and ethyl acetate. The organic layer was separated and the aqueous layer was extracted with ethyl acetate. The combined organic layer was washed with brine and dried over anhydrous MgSO_4_, filtrated and concentrated *in vacuo*, the residue was purified through column chromatography on silica gel to give the desired products.

## Conflicts of interest

There are no conflicts to declare.

## Supplementary Material

RA-012-D2RA00241H-s001
