# Supplementary material for: Transient directing group enabled Pd-catalyzed C–H oxygenation of benzaldehydes and benzylic amines
Source: RSC Adv. 2022 Jun 27;12(29):18722–7. doi: 10.1039/d2ra00241h (PMC9235058; doi:10.1039/d2ra00241h)

Electronic Supplementary Material (ESI) for RSC Advances.
This journal is © The Royal Society of Chemistry 2022

Supporting Information

Transient Directing Group Enabled Pd-catalyzed C–H Oxygenation of Benzaldehydes and Benzylic Amines

Mixiang Tian,[a] Lidong Shao,[a] Xiaosan Su,[a] Xuhong Zhou,[a] Honglei Zhang,[a] Kun Wei,[b] Ruifen Sun*[a] and Junliang Wang*[a]

[a] Center for scientific research, Yunnan University of Chinese Medicine, Kunming, 650500 (P. R. China). E-mail: wangjunliangedu@163.com; sunruifen@ynutcm.edu.cn.

[b] School of Chemical Science and Technology, Yunnan University, Kunming, 650091 (P. R. China).

Table of Contents

General Information 1

Experimental Section 1

S1. Optimization of the fluoroalkoxylation of benzaldehydes 2

S2. General Procedure for *ortho*-C□H polyfluoroalkoxylation of benzaldehydes 4

S3. Optimization of the consecutive oxidation of benzylamines..5

S4. Typical Procedure for the C–N and *ortho*-C(sp^2)–H consecutive oxidation of benzylamines 6

S5 Comparison of turnover frequency of different systems 7

The Characterization of Reaction Products..8

Reference…………………………………… … ……….16

Copies of NMR spectra of compounds 17

General Information

Unless otherwise noted, all the reagents (including all fluorinated alcohols are known compounds), starting materials and ligands (**A1-A9**) were purchased from commercial vendors (e. g. Adamas-beta) and used without further purification. Reactions were monitored by thin layer chromatography (TLC) carried out on 250 µm Merck silica gel plates (60 F254) containing a fluorescent indicator (254 nm). Visualization of the developed TLC plate was performed by irradiation with UV light. NMR spectra were recorded on a Bruker Ascend 500 spectrometer at 500 MHz (^1H NMR), 125 MHz (^{13}C NMR) and are referenced relative to residual $CHCl_3$ (in $CDCl_3$) proton signals at δ 7.26 ppm. Data for ^1H spectra are reported as follows: chemical shift (δ ppm), multiplicity (s = singlet, d = doublet, t = triplet, q = quartet, m = multiplet, b = broad, ap = apparent), integration, coupling constant (Hz) and assignment. ^{13}C NMR spectra were recorded on a Bruker 500 (500 MHz) and are referenced relative to residual $CHCl_3$ (in $CDCl_3$) at δ 77.16 ppm. Data for ^{13}C NMR spectra are reported in terms of chemical shift and multiplicity where appropriate. All known products gave satisfactory analytical data by ^1H NMR or ^{13}C NMR spectra, corresponding to the reported literature values.[1-4]

Experimental Section

S1 Optimization of the fluoroalkoxylation of benzaldehydes

An 8-mL vial equipped with a stir bar was charged with $Pd(OAc)_2$ (4.5 mg, 0.02 mmol, 10 mol %), ligands **A** (0.1 mmol, 50 mol%), Oxidants (0.4 mmol, 2.0 equiv.), and benzaldehyde (0.2 mmol, 1.0 equiv.), followed by the addition of fluorinated alcohol (2.0 mL) and TFA(2.0 equiv.). The flask was then sealed and the mixture was stirred at room temperature for 10 min before being heated to 80 °C for 12 h. The reaction mixture was cooled to room temperature, followed by the addition of water and ethyl acetate. The organic layer was separated and the aqueous layer was extracted with ethyl acetate. The combined organic layer was

washed with brine and dried over anhydrous $MgSO_4$, filtrated and concentrated in vacuo, the residue was purified through column chromatography on silica gel.

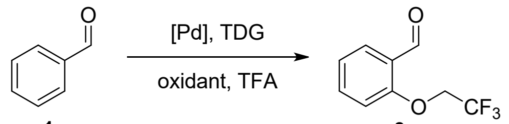

1 **3**

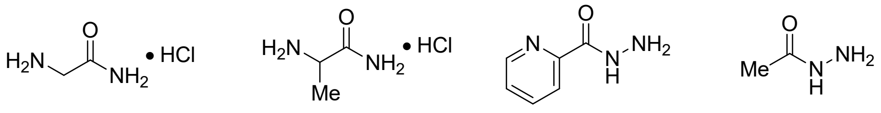

A1 **A2** **A3** **A4**

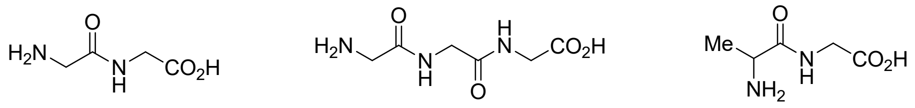

A5 **A6** **A7**

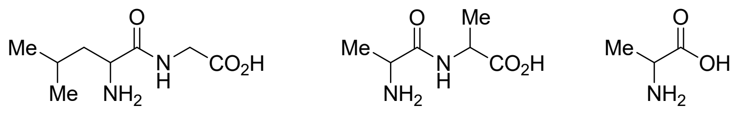

A8 **A9** **A10**

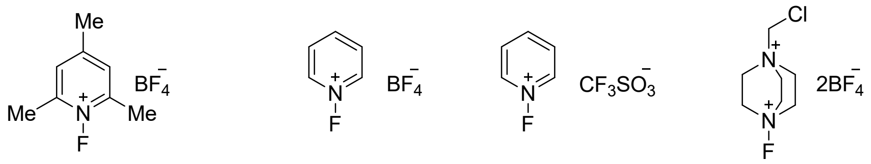

F1 **F2** **F3** **F4**

Entry	Oxidant	TDG (50 mol %)	TFA (equiv.)	Yield (%) [b]
1	$K_2S_2O_8$	**A1**	2	30
2	$K_2S_2O_8$	**A2**	2	30
3	$K_2S_2O_8$	**A3**	2	0
4	$K_2S_2O_8$	**A4**	2	20
5	$K_2S_2O_8$	**A5**	2	trace
6	$K_2S_2O_8$	**A6**	2	30
7	$K_2S_2O_8$	**A7**	2	25
8	$K_2S_2O_8$	**A8**	2	20

9	$K_2S_2O_8$	**A9**	2	60
10	**F1**	**A9**	2	88
11	**F2**	**A9**	2	60
12	**F3**	**A9**	2	45
13	**F4**	**A9**	2	60
14	other oxidants*	**A9**	2	<30
15	**F1**	**A9**	0	30
16	**F1**	**A9**	1	50
17	**F1**	**A9**	3	80
18	**F1**	**A9**	5	78
19	**F1**	**A9**	10	78
20[c]	**F1**	**A9**	2	75
21[d]	**F1**	**A9**	2	60
22[e]	**F1**	**A9**	2	trace
23[f]	**F1**	**A9**	2	30
24[g]	**F1**	**A9**	2	trace
25[h]	**F1**	**A9**	2	trace
26[i]	**F1**	**A9**	2	25
27	**F1**	**A10**	2	35
28	**F1**	**A9** (30 mol %)	2	30
29	**F1**	**A9**(70 mol %)	2	72
30	**-**	**A9**	2	N.R.
31[j]	**F1**	**A9**	2	N.R.

[a] reaction conditions: 1a (0.2 mmol), TFE (2 mL), [Pd] (10 mol %), TDG (50 mol %), oxidant (0.4 mmol), TFA (2 equiv.) and stirred at 80 °C for 12 h. [b] Isolated yield. Other oxidants*:

$(NH_4)_2S_2O_8$, $Na_2S_2O_8$, $PhI(OAc)_2$, AgTFA, AgOAc, Ag_2CO_3, Ag_2O. [c] 60°C. [d] 40 °C. [e] solvent: TFE/Toluene (v/v, 1 : 1). [f] solvent: TFE/DCM (v/v, 1 : 1). [g] DCM as solvent, 10eq TFE. [h] DCM as solvent, 20eq TFE. [I] DCM as solvent, 30eq TFE. [j] without $Pd(OAc)_2$.

S2 General Procedure for *ortho*-C–H polyfluoroalkoxylation of benzaldehydes

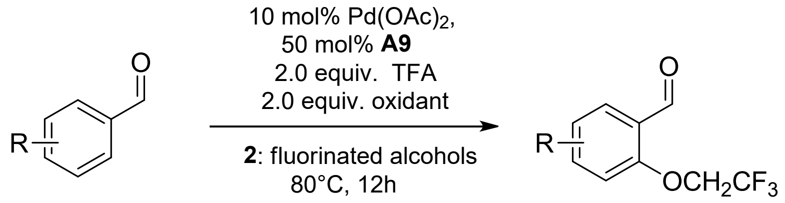

1 **3**

An 8-mL vial equipped with a stir bar was charged with $Pd(OAc)_2$ (4.5 mg, 0.02 mmol, 10 mol %), **A9** (16.0 mg, 0.1 mmol, 50 mol%), 1-fluoro-2,4,6-trimethylpyrdinium triflate (F1) (90.8 mg, 0.4 mmol, 2.0 equiv.), and benzaldehydes (0.2 mmol, 1.0 equiv.), followed by the addition of fluorinated alcohol (2.0 mL) and TFA (2.0 equiv.). The flask was then sealed and the mixture was stirred at room temperature for 10 min before being heated to 80 °C for 12 h. The reaction mixture was cooled to room temperature, followed by the addition of water and ethyl acetate. The organic layer was separated and the aqueous layer was extracted with ethyl acetate. The combined organic layer was washed with brine and dried over anhydrous $MgSO_4$, filtrated and concentrated in vacuo, the residue was purified through column chromatography on silica gel to give the desired products.

S3 Optimization of the consecutive oxidation of benzylamines

Oxidants (0.4 mmol, 2.0 equiv.) were added to a solution of benzylamines (0.2 mmol, 1.0 equiv.) in fluorinated alcohol (2.0 mL). The reaction mixture was heated and stired for 30 min, followed by the addition of $Pd(OAc)_2$ (4.5 mg, 0.02 mmol, 10 mol %), **A9** (16.0 mg, 0.1 mmol, 50 mol%), and TFA (2.0 equiv.). The reaction mixture was stirred at the same temperature for 12 h. The reaction mixture was cooled to room temperature, followed by the addition of water and ethyl acetate. The organic layer was separated and the aqueous layer was extracted with ethyl acetate. The combined organic layer was washed with brine and dried over anhydrous $MgSO_4$, filtrated and concentrated in vacuo, the residue

was purified through column chromatography on silica gel.

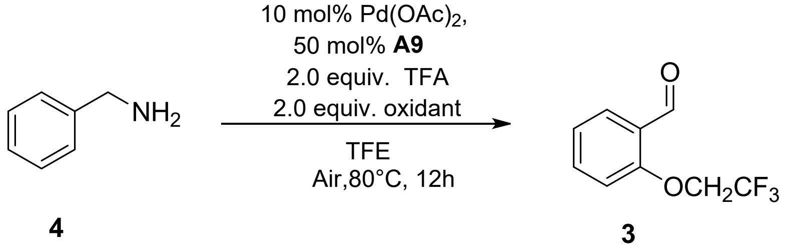

Entry	Oxidant	Temperature(℃)	Yield (%) [a]
1	**F1**	80	18%
2	$K_2S_2O_8$	80	70%
3	**F2**	80	trace
4	**F3**	80	trace
5	**F4**	80	10% (86%) [b]
6[c]	**F1**+ $K_2S_2O_8$	80	78%
7[d]	**F1**+**F4**	80	80%
8[d]	**F1**+**F4**	60	70%
9[d]	**F1**+**F4**	50	55%
10[e]	**F1**+**F4**	80	78%

[a] Isolated yields; [b] Benzaldehyde was formed as major product; [c] **F1** (0.2 mmol), $K_2S_2O_8$ (0.2 mmol), under air; [d] **F1** (0.2 mmol), F4 (0.2 mmol), under air; [e] **F1** (0.2 mmol), F4 (0.2 mmol), under N_2.

S4 Typical Procedure for the C–N and *ortho*-C(sp[2])–H consecutive oxidation of benzylamines

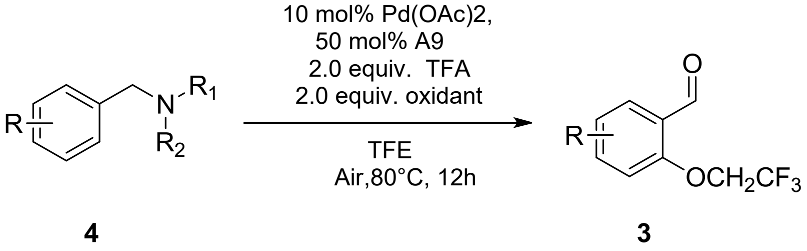

Selectfluor (**F4**) (70.8 mg, 0.2 mmol, 1.0 equiv.) and 1-fluoro-2,4,6-trimethylpyrdinium triflate (**F1**) (90.8 mg, 0.2 mmol, 1.0 equiv.) were added to a solution of benzylamines (0.2 mmol, 1.0 equiv.) in TFE (2.0 mL). The reaction mixture was stirred at 80 °C for 30 min under air, followed by the addition of $Pd(OAc)_2$ (4.5 mg, 0.02 mmol, 10 mol %), **A9** (16.0 mg, 0.1 mmol, 50 mol%), and TFA (2.0 equiv.). The reaction mixture was stirred at the same temperature for 12 h. Upon completion, the reaction mixture was cooled to room temperature, followed by the addition of water and ethyl acetate. The organic layer was separated and the aqueous layer was extracted with ethyl acetate. The combined organic layer was washed with brine and dried over anhydrous $MgSO_4$, filtrated and concentrated in vacuo, the residue was purified through column chromatography on silica gel to give the desired products.

S5 Comparison of turnover frequency of different systems

Comparing the turnover frequency of **A9**-[F^+]-Pd-benzaldehyde systems with other Pd-TDG-benzaldehyde systems,[1,4] we found that the conversion efficiency of **A9**-[F^+]-Pd-benzaldehyde system is better, and a certain amount of transformation can be achieved within 15 hours (Figure 1).

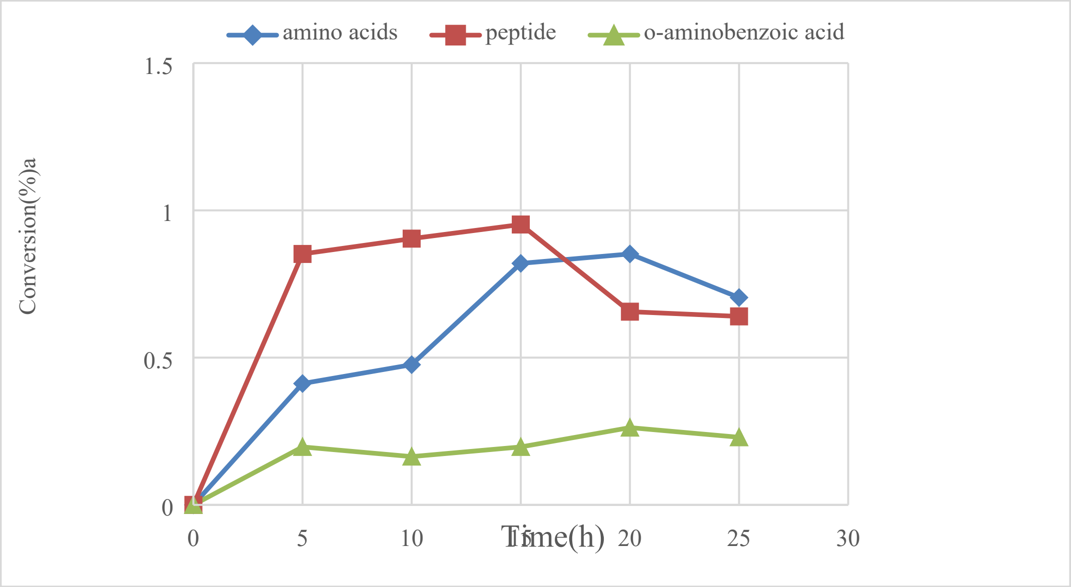

[a] Conversion were determined by ^1H NMR analysis of the crude product using 1,3-benzodioxole as the internal standard.

■: Pd/F1/peptide system, ◆: Pd/$K_2S_2O_8$/amino acid system, ▲: Pd/2-amino-5-chlorobenzoic acid system.

Figure 1

The Characterization of Reaction Products

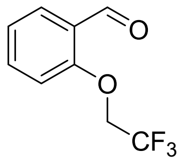

2-(2,2,2-Trifluoroethoxy)benzaldehyde. (3a)

This compound was prepared by the general procedure described above and was obtained as white solid (12 h, 26 mg, 88%). R_f (PE : EtOAc = 10:1): 0.3, [known compound[1,2]]; ^{1}H NMR (500 MHz, $CDCl_3$) δ 10.49 (s, 1H), 7.90 (dd, J = 7.7, 1.7Hz, 1H), 7.59 (m, 1H), 7.18 (t, J = 7.5 Hz, 1H), 6.98 (d, J = 8.4 Hz, 1H), 4.50 (q, J = 7.9 Hz, 2H). ^{13}C NMR (500 MHz, $CDCl_3$) δ 188.8, 159.4, 135.9, 128.8, 125.6, 122.8 (C-F, $^1J_{C\text{-}F}$ = 278.1 Hz), 122.9, 113.0, 66.3 (C-F, $^2J_{C\text{-}F}$ = 36.1 Hz).

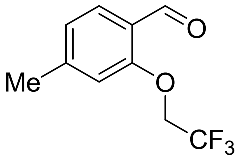

4-Methyl-2-(2,2,2-trifluoroethoxy)benzaldehyde. (3b)

This compound was prepared by the general procedure described above and was obtained as white solid (12 h, 22 mg, 62%). R_f (PE : EtOAc = 10:1): 0.3, [known compound[1]]; ^{1}H NMR (500 MHz, $CDCl_3$) δ 10.44 (d, J = 0.65 Hz,1H), 7.81 (d, J = 7.9 Hz, 1H), 6.99 (d, J = 7.9 Hz, 1H), 6.77 (s, 1H), 4.5 (q, J = 8.0 Hz, 2H), 2.44 (s, 3H). ^{13}C NMR (500 MHz, $CDCl_3$) δ 188.5, 159.4, 147.5, 128.8, 123.7, 123.4, 122.0 (C-F, $^1J_{C\text{-}F}$ = 278.6Hz), 113.5, 66.4 (C-F, $^2J_{C\text{-}F}$ = 36.1Hz),22.2.

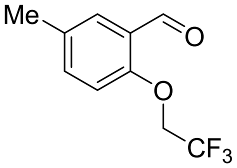

5-Methyl-2-(2,2,2-trifluoroethoxy)benzaldehyde. (3c)

This compound as prepared by the general procedure described above and was obtaines white solid (12 h, 24 mg, 65%). R_f (PE : EtOAc = 10:1): 0.3, [known compound[1]]; ^{1}H NMR (500 MHz, $CDCl_3$) δ 10.48 (s, 1H), 7.7 (d, J = 2.1 Hz, 1H), 7.39(dd, J = 2.2, 8.6 Hz, 1H), 6.89 (d, J = 8.5 Hz, 1H), 4.47 (q, J = 8.0 Hz, 2H), 2.36 (s, 3H). ^{13}C NMR (500 MHz, $CDCl_3$) δ 189.0, 157.5, 136.4, 132.5,128.9, 125.3, 124.2 (C-F, $^1J_{C-F}$ = 278.0 Hz), 113.0, 66.9 (C-F, $^2J_{C-F}$ = 35.9 Hz)，20.3.

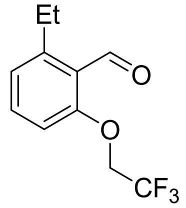

2-Ethyl-6-(2,2,2-trifluoroethoxy)benzaldehyde. (3d)

This compound was prepared by the general procedure described above and was obtained as white solid(12 h, 24 mg, 56%). R_f (PE : EtOAc = 10:1): 0.3, [known compound[1]]; ^{1}H NMR (500 MHz, $CDCl_3$) δ 10.64 (d, J = 5.0 Hz, 1H), 7.45 (t, J = 6.7 Hz, 1H), 6.69 (d, J = 6.4 Hz, 1H), 6.81 (d, J = 6.4 Hz, 1H), 4.46 (d, J = 7.0 Hz, 2H), 3.0 (d, J = 6.7 Hz, 2H), 1.27 (d, J = 6.4 Hz, 3H). ^{13}C NMR (500 MHz, $CDCl_3$) δ191.3, 160.7, 148.8, 134.6, 124.6, 123.7, 121.7 (C-F, $^1J_{C-F}$ = 278.1 Hz),110.3, 66.6(C-F, $^2J_{C-F}$ = 36.1 Hz), 26.9, 15.4.

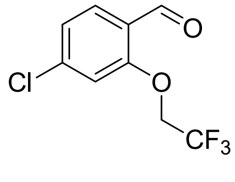

4-Chloro-2-(2,2,2-trifluoroethoxy)benzaldehyde. (3e)

This compound was prepared by the general procedure described above and was obtained as white solid (12 h, 24 mg, 52%). R_f (PE : EtOAc = 10:1): 0.3, [known compound[1]]; ^{1}H NMR (500 MHz, $CDCl_3$) δ 10.42 (d, J = 0.6 Hz, 1H), 7.86 (d, J = 8.3 Hz, 1H), 7.18 (dd, J = 8.0, 1.2 Hz, 1H), 7.0 (d, J = 1.5 Hz, 1H), 4.52 (q, J = 7.8 Hz, 2H). ^{13}C NMR (500MHz, $CDCl_3$) δ 187.5, 159.5, 142.0, 130.0, 124.1, 123.9, 121.7 (C-F, $^1J_{C-F}$ = 278.0 Hz), 113.6, 66.5 (C-F, $^2J_{C-F}$ = 36.5 Hz).

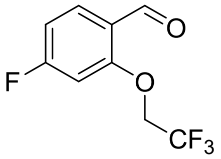

4-Fluoro-2-(2,2,2-trifluoroethoxy)benzaldehyde. (3f)

This compound was prepared by the general procedure described above and was obtained as white solid (12 h, 25 mg, 52%). R_f (PE : EtOAc = 10:1): 0.3, [known compound[1]]; ^{1}H NMR (500 MHz, $CDCl_3$) δ 10.4 (s, 1H), 7.95 (dd, *J* = 8.7, 6.7 Hz, 1H), 6.9 (td, *J* = 8.3, 2.1 Hz, 1H), 6.7 (dd, *J* =9.8, 2.2 Hz, 1H), 4.50 (q, *J* = 7.8 Hz, 2H). ^{13}C NMR (500 MHz, $CDCl_3$) δ 187.2, 168.3(C-F, $^1J_{C\text{-}F}$ = 257.6 Hz) , 160.8 (C-F, $^3J_{C\text{-}F}$ = 10.7 Hz), 131.3 (C-F, $^3J_{C\text{-}F}$ = 11.3 Hz), 123.9(C-F, $^1J_{C\text{-}F}$ = 278.0 Hz),122.3(C-F, $^4J_{C\text{-}F}$ = 3.0 Hz), 110.2(C-F, $^2J_{C\text{-}F}$ = 21.9 Hz),101.0 (C-F, $^2J_{C\text{-}F}$ = 26.2 Hz), 66.3 (C-F, $^2J_{C\text{-}F}$ = 36.5 Hz).

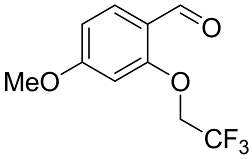

4-Methoxy-2-(2,2,2-trifluoroethoxy)benzaldehyde. (3g)

This compound was prepared by the general procedure described above and was obtained as white solid (12 h, 18 mg, 73%). R_f (PE : EtOAc = 10:1): 0.3, [known compound[1]]; ^{1}H NMR (500 MHz, $CDCl_3$) δ 10.31 (s, 1H), 7.87 (d, *J* = 8.7 Hz, 1H), 6.66 (dd, *J* = 8.7, 2.0 Hz, 1H), 6.41 (d, *J* = 1.9 Hz, 1H), 4.45 (q, *J* = 7.9 Hz, 2H), 3.88 (s, 3H). ^{13}C NMR (500 MHz, $CDCl_3$) δ 187.5, 166.0, 161.0, 131.0,124.1 (C-F, 1$J_{C\text{-}F}$ = 278.0 Hz), 119.6, 107.6, 99.5, 66.3 (C-F, 2$J_{C\text{-}F}$ =36.1 Hz), 55.8.

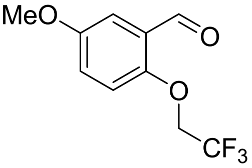

5-Methoxy-2-(2,2,2-trifluoroethoxy)benzaldehyde. (3h)

This compound was prepared by the general procedure described above and was obtained as white solid (12 h, 29 mg, 50%). R_f (PE : EtOAc = 10:1): 0.3, [known compound[1]]; ^{1}H NMR (500 MHz, $CDCl_3$) δ 10.45(s, 1H), 7.37 (d, J = 3.24 Hz, 1H), 7.17 (d, J = 3.26 Hz, 1H), 6.96 (d, J =9.04 Hz, 1H), 4.45 (q, J = 8.0 Hz, 2H), 3.84 (s, 3H). ^{13}C NMR (500 MHz, $CDCl_3$) δ 188.7, 155.2, 154.1, 132.0,123.2 (C-F, $^1J_{C-F}$ = 278.0 Hz), 115.4, 114.3,110.9, 77.1 (C-F, $^2J_{C-F}$ =32.0 Hz), 55.8.

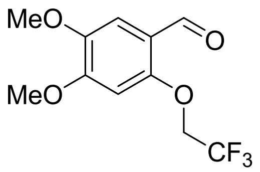

4,5-Dimethoxy-2-(2,2,2-trifluoroethoxy)benzaldehyde. (3i)

This compound was prepared by the general procedure described above and was obtained as white solid (12 h, 32 mg, 80%). R_f(PE : EtOAc = 10:1): 0.3; ^{1}H NMR (500 MHz, $CDCl_3$) δ 10.33 (s, 1H), 7.35(s, 1H), 6.5 (s, 1H), 6.41 (d, J = 1.9 Hz, 1H), 4.5(q, J = 8.0Hz, 2H), 3.98 (s, 3H), 3.91 (s, 3H). ^{13}C NMR (500 MHz, $CDCl_3$) δ 187.3, 155.9, 155.6, 145.3,124.1 (C-F, $^1J_{C-F}$ = 278.0 Hz), 118.8, 109.1, 98.3, 77.3 (C-F, $^2J_{C-F}$ =36.1 Hz), 56.3.

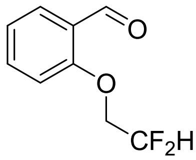

2-(2,2-Difluoroethoxy)benzaldehyde. (3l)

This compound was prepared by the general procedure described above and was obtained as white solid (12 h, 23 mg, 70%). R_f (PE : EtOAc = 10:1): 0.3, [known compound[1]]; ^{1}H NMR (400 MHz, $CDCl_3$) δ 10.5 (s, 1H), 7.88 (dd,J = 7.7,1.8Hz, 1H), 7.61 – 7.58 (m, 1H), 7.12 (d, J = 8.4 Hz, 1H), 6.97 (d, J = 8.4 Hz, 1H), 6.97 (td, J = 25.43, 50.86 Hz, 1H 1H), 4.36 – 4.3 (m, 2H).

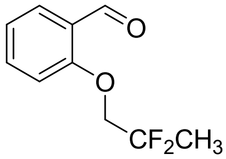

2-(2,2-Difluoropropoxy)benzaldehyde. (3m)

This compound was prepared by the general procedure described above and was obtained as white solid (12 h, 20 mg, 60%). R_f (PE : EtOAc = 10:1): 0.3, [known compound[1]]; ^{1}H NMR (500 MHz, $CDCl_3$) δ 10.53 (s, 1H), 7.89 (dd, J = 7.7,1.8 Hz, 1H), 7.67–7.57 (m, 1H), 7.01 (dd, J = 19.0, 8.4 Hz, 2H), 4.26 (t, J = 11.13 Hz, 2H), 1.83 (t, J = 9.4 Hz, 3H).

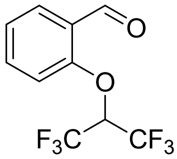

2-((1,1,1,3,3,3-Hexafluoropropan-2-yl)oxy)benzaldehyde. (3n)

This compound was prepared by the general procedure described above and was obtained as white solid (12 h, 18 mg, 80%). R_f (PE : EtOAc = 10:1): 0.3, [known compound[1, 4]]; ^{1}H NMR (500 MHz, $CDCl_3$) δ 10.49 (s, 1H), 7.95 (dd, J = 7.7, 1.7 Hz, 1H), 7.68–7.65 (m, 1H), 7.32–7.28 (m, 1H), 7.12 (d, J = 8.4 Hz, 1H), 5.07–5.04(m, 1H).

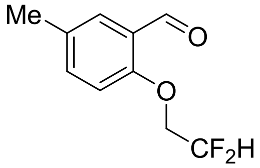

2-(2,2-Difluoroethoxy)-5-methylbenzaldehyde. (3o)

This compound was prepared by the general procedure described above and was obtained as Yellow oil (12 h, 24 mg, 53%). R_f (PE : EtOAc = 10:1): 0.3, [known compound[1]]; ^{1}H NMR (500 MHz, $CDCl_3$) δ 10.45 (s, 1H), 7.66 (d, J = 2.2 Hz, 1H), 7.38 (dd, J = 8.7, 2.1 Hz, 1H), 6.87 (d, J = 8.5 Hz, 1H), 6.15–6.01 (m, J = 54.9, 4.1 Hz,

1H), 4.3 (td, J = 13.0, 4.1 Hz, 2H), 2.33 (s, 3H). ^{13}C NMR (101 MHz, $CDCl_3$) δ 189.2, 158.0, 136.5, 131.8, 128.9, 125.0, 113.3 (C-F, $^1J_{C-F}$ = 241.5 Hz), 112.7, 67.8 (C-F, $^2J_{C-F}$ = 29.6 Hz), 20.3.

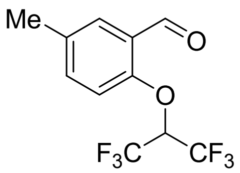

2-((1,1,1,3,3,3-Hexafluoropropan-2-yl)oxy)-5-methylbenzaldehyde. (3p)

This compound was prepared by the general procedure described above and was obtained as white solid (12 h, 20 mg, 49%). R_f (PE : EtOAc = 10:1): 0.3, [known compound[1]]; ^{1}H NMR (500 MHz, $CDCl_3$) δ 10.48 (s, 1H), 7.69 (d, J = 2.2 Hz, 1H), 7.39 (dd, J = 8.7, 2.1 Hz, 1H), 6.89 (d, J = 8.5 Hz, 1H), 6.17–6.05 (m, J = 54.9, 4.1 Hz, 1H), 4.3 (td, J = 13.0, 4.1 Hz, 2H), 2.36 (s, 3H).

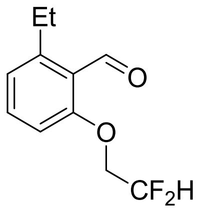

2-(2,2-Difluoroethoxy)-6-ethylbenzaldehyde. (3q)

This compound was prepared by the general procedure described above and was obtained as Yellow oil (12 h, 52 mg, 64%). R_f (PE : EtOAc = 10:1): 0.3; ^{1}H NMR (500 MHz, $CDCl_3$) δ 10.65 (s, 1H), 7.45 (t, J = 8.03 Hz, 1H), 6.83 (d, J = 8.4 Hz 1H), 6.97 (d, J = 7.8 Hz, 1H), 6.17 (ddd, J = 54.9,29.5,4.1 Hz, 1H), 4.32 – 4.26 (m, 2H),3.0 (q, J = 7.5 Hz, 2H), 1.2 (q, J = 7.5 Hz, 3H). ^{13}C NMR (500 MHz, $CDCl_3$) δ 191.5, 161.1, 148.7, 134.6, 124.0, 123.4 (C-F, $^1J_{C-F}$ = 241.6 Hz), 113.3, 109.8, 67.8 (C-F, $^2J_{C-F}$ = 29.7 Hz), 26.9, 15.5.

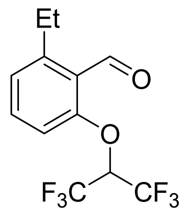

1-ethyl-6-((1,1,1,3,3,3-Hexafluoropropan-2-yl)oxy)benzaldehyde. (3r)

This compound was prepared by the general procedure described above and was obtained as white solid (12 h, 21 mg, 55%). R_f (PE : EtOAc = 10:1): 0.3, [known compound[4]]; ^{1}H NMR (500 MHz, $CDCl_3$) δ 10.63 (s, 1H), 7.53 (t, J = 8.06 Hz 1H), 7.12 (d, J = 7.76 Hz, 1H), 6.94 (d, J = 8.4 Hz, 1H), 5.01 (dt, J = 11.2, 5.6 Hz, 1H), 3.02 (q, J = 7.5, 2H), 1.24 (t, J = 7.5 Hz, 3H).

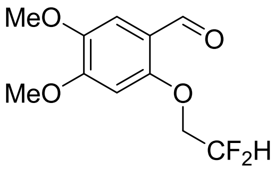

2-(2,2-Difluoroethoxy)-4,5-dimethoxybenzaldehyde. (3s)

This compound was prepared by the general procedure described above and was obtained as Yellow solid (12 h, 24 mg, 56%). R_f (PE : EtOAc = 10:1): 0.3; ^{1}H NMR (500 MHz, $CDCl_3$) δ 10.32 (s, 1H), 7.33 (d, J = 3.6, 2.3 Hz, 1H), 6.49 (s, 1H) , 6.15 (tt, J = 54.9,4.0 Hz, 1H), 4.3 (td, J = 13.0, 4.0 Hz, 2H), 3.97 (d, J = 40.3 Hz, 6H).

The NMR data of several compounds were randomly selected and compared with the data in references.

compounds	^{1}H	^{13}C
3a	δ 10.49 (s, 1H), 7.90 (dd, *J* = 7.7, 1.7Hz, 1H), 7.59 (m, 1H), 7.18 (t, *J* = 7.5 Hz, 1H), 6.98 (d, *J* = 8.4 Hz, 1H), 4.50 (q, *J* = 7.9 Hz, 2H).	δ 188.8, 159.4, 135.9, 128.8, 125.6, 122.8 (C-F, $^1J_{C\text{-}F}$ = 278.1 Hz), 122.9, 113.0, 66.3 (C-F, $^2J_{C\text{-}F}$ = 36.1 Hz)
Reference1	δ 10.49 (d, *J* = 0.7 Hz, 1H), 7.88 (dd, *J* = 7.7, 1.8 Hz, 1H), 7.59 (ddd, *J* = 8.4, 7.4, 1.8 Hz, 1H), 7.16 (t, *J* = 7.5 Hz, 1H), 6.97 (d, *J* = 8.4 Hz, 1H), 4.50 (q, *J* = 7.9 Hz, 2H).	δ 188.9, 159.5, 136.0, 128.9, 125.7, 123.2 (C-F, $^1J_{C\text{-}F}$ = 278.1 Hz), 122.9, 113.0, 66.3 (C-F, $^2J_{C\text{-}F}$ = 36.1 Hz).
3b	δ 10.44 (d, *J* = 0.65 Hz,1H), 7.81 (d, *J* = 7.9 Hz, 1H), 6.99 (d, *J* = 7.9 Hz, 1H), 6.77 (s, 1H), 4.5 (q, *J* = 8.0 Hz, 2H), 2.44 (s, 3H).	δ 188.5, 159.4, 147.5, 128.8, 123.7, 123.4, 122.0 (C-F, $^1J_{C\text{-}F}$ = 278.6Hz), 113.5, 66.4 (C-F, $^2J_{C\text{-}F}$ = 36.1Hz),22.2.
Reference1	δ 10.42 (s, 1H), 7.78 (d, *J* = 7.9 Hz, 1H), 6.96 (d, *J* = 7.9 Hz, 1H), 6.75 (s, 1H), 4.47 (q, *J* = 7.9 Hz, 2H), 2.42 (s, 3H).	δ 188.6, 159.6, 147.7, 128.9, 123.9, 123.5, 123.2 (C-F, $^1J_{C\text{-}F}$ = 278.0 Hz), 113.6, 66.3 (C-F, $^2J_{C\text{-}F}$ = 36.1 Hz), 22.3.
3e	δ 10.42 (d, *J* = 0.6 Hz, 1H), 7.86 (d, *J* = 8.3 Hz, 1H), 7.18 (dd, *J* = 8.0, 1.2 Hz, 1H), 7.0 (d, *J* = 1.5 Hz, 1H), 4.52 (q, *J* = 7.8 Hz, 2H).	δ 187.5, 159.5, 142.0, 130.0, 124.1, 123.9, 121.7 (C-F, $^1J_{C\text{-}F}$ = 278.0 Hz), 113.6, 66.5 (C-F, $^2J_{C\text{-}F}$ = 36.5 Hz).
Reference1	δ 10.40 (s, 1H), 7.83 (d, *J* = 8.3 Hz, 1H), 7.14 (dd, *J* = 8.3, 0.8 Hz, 1H), 6.99 (d, *J* = 1.5 Hz, 1H), 4.50 (q, *J* = 7.8 Hz, 2H).	δ 187.7, 159.7, 142.0, 130.1, 124.2, 123.5, 122.9 (C-F, $^1J_{C\text{-}F}$ = 278.0 Hz), 113.7, 66.5 (C-F, $^2J_{C\text{-}F}$ = 36.5 Hz).

3f	δ 10.4 (s, 1H), 7.95 (dd, J = 8.7, 6.7 Hz, 1H), 6.9 (td, J = 8.3, 2.1 Hz, 1H), 6.7 (dd, J =9.8, 2.2 Hz, 1H), 4.50 (q, J = 7.8 Hz, 2H).	δ 187.2, 168.3(C-F, $^{1}J_{C\text{-}F}$ = 257.6 Hz) , 160.8 (C-F, $^{3}J_{C\text{-}F}$ = 10.7 Hz), 131.3 (C-F, $^{3}J_{C\text{-}F}$ = 11.3 Hz), 123.9(C-F, $^{1}J_{C\text{-}F}$ = 278.0 Hz),122.3(C-F, $^{4}J_{C\text{-}F}$ = 3.0 Hz), 110.2(C-F, $^{2}J_{C\text{-}F}$ = 21.9 Hz),101.0 (C-F, $^{2}J_{C\text{-}F}$ = 26.2 Hz), 66.3 (C-F, $^{2}J_{C\text{-}F}$ = 36.5 Hz).
Reference1	δ 10.37 (s, 1H), 7.91 (dd, J = 8.7, 6.7 Hz, 1H), 6.86 (td, J = 8.1, 1.7 Hz, 1H), 6.71 (dd, J = 10.0, 2.2 Hz, 1H), 4.50 (q, J = 7.8 Hz, 2H).	δ 187.4, 167.4 (C-F, $^{1}J_{C\text{-}F}$ = 257.6 Hz), 160.9 (C-F, $^{3}J_{C\text{-}F}$ = 10.8 Hz), 131.3 (C-F, $^{3}J_{C\text{-}F}$ = 11.3 Hz), 122.9 (C-F, $^{1}J_{C\text{-}F}$ = 278.0 Hz), 122.4 (C-F, $^{4}J_{C\text{-}F}$ = 3.0 Hz), 110.3 (C-F, $^{2}J_{C\text{-}F}$ = 21.9 Hz), 101.1 (C-F, $^{2}J_{C\text{-}F}$ = 26.2 Hz), 66.4 (C-F, $^{2}J_{C\text{-}F}$ = 36.4 Hz)

Reference

1 L. Jin, X.-L. Zhang, R.-L. Guo, M.-Y. Wang, Y.-R. Gao, and Y.-Q. Wang, *Org. Lett.*, 2021, **23**, 1921−1927.

2 B. Pethő, M. Zwillinger, J. T. Csenki, A. E. Káncz, B. Krámos, J. Müller, G. T. Balogh, Z. Novák, *Chemistry – A European Journal*, 2017, **23**, 15628-15632.

3 X. Chen, S. Ozturk, E. J. Sorensen, *Organic Letters*, 2017, **19**, 6280-6283.

4 J. Zhou, D. Liu, C. Bai, A. Bao, T. Muschin, M. Baiyin and Y. S. Bao, *Org. Chem. Front.*, 2021, **8**, 5975-5981.

Copies of NMR spectra of compounds

^{1}H NMR (500 MHz, $CDCl_3$) spectrum of compound **3a**

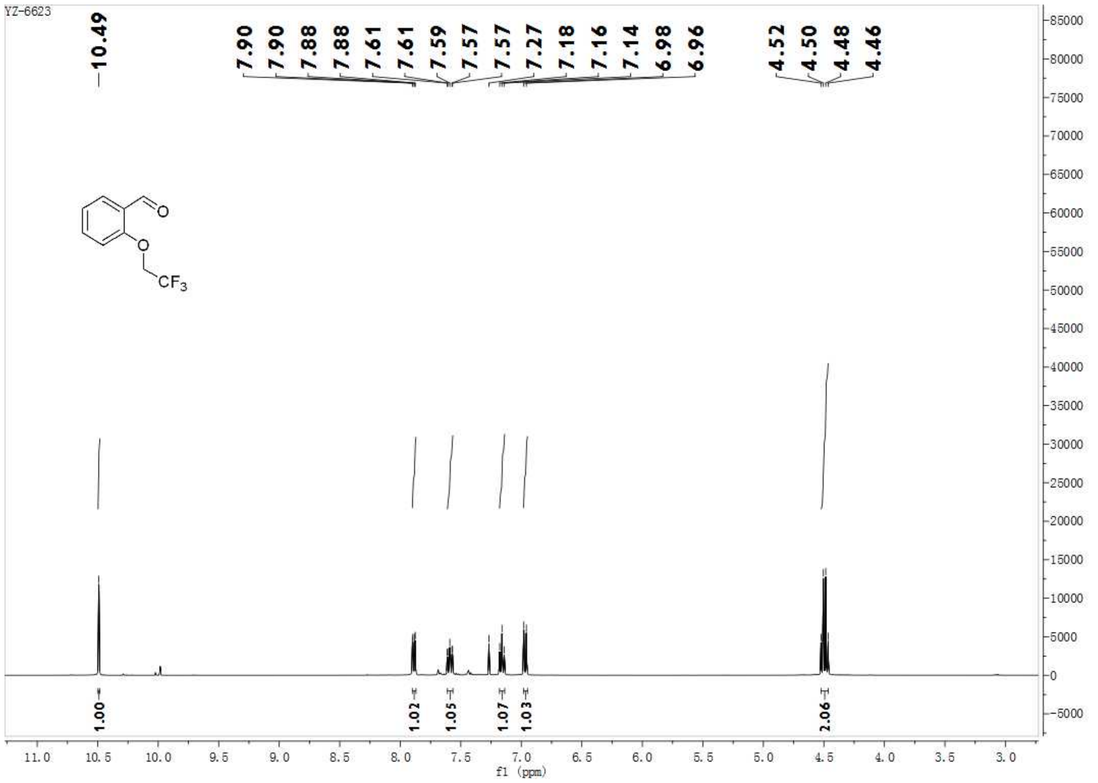

^{13}C NMR (500 MHz, $CDCl_3$) spectrum of compound **3a**

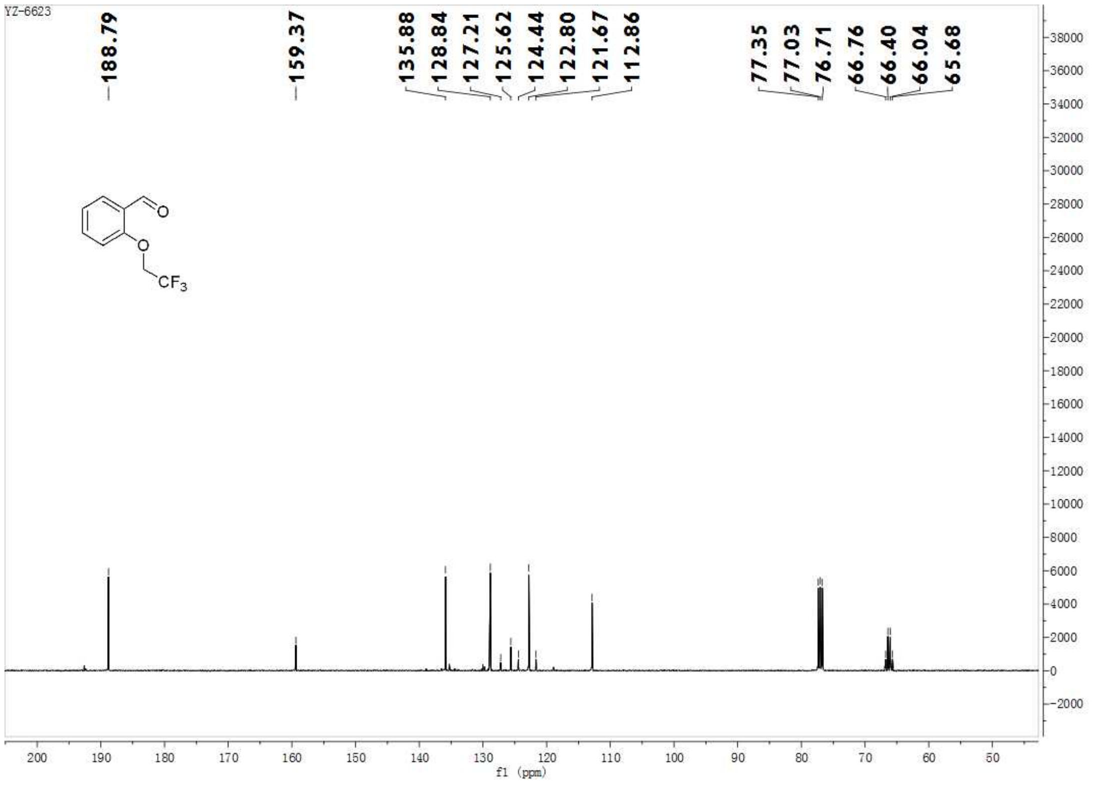

^{1}H NMR (500 MHz, $CDCl_3$) spectrum of compound **3b**

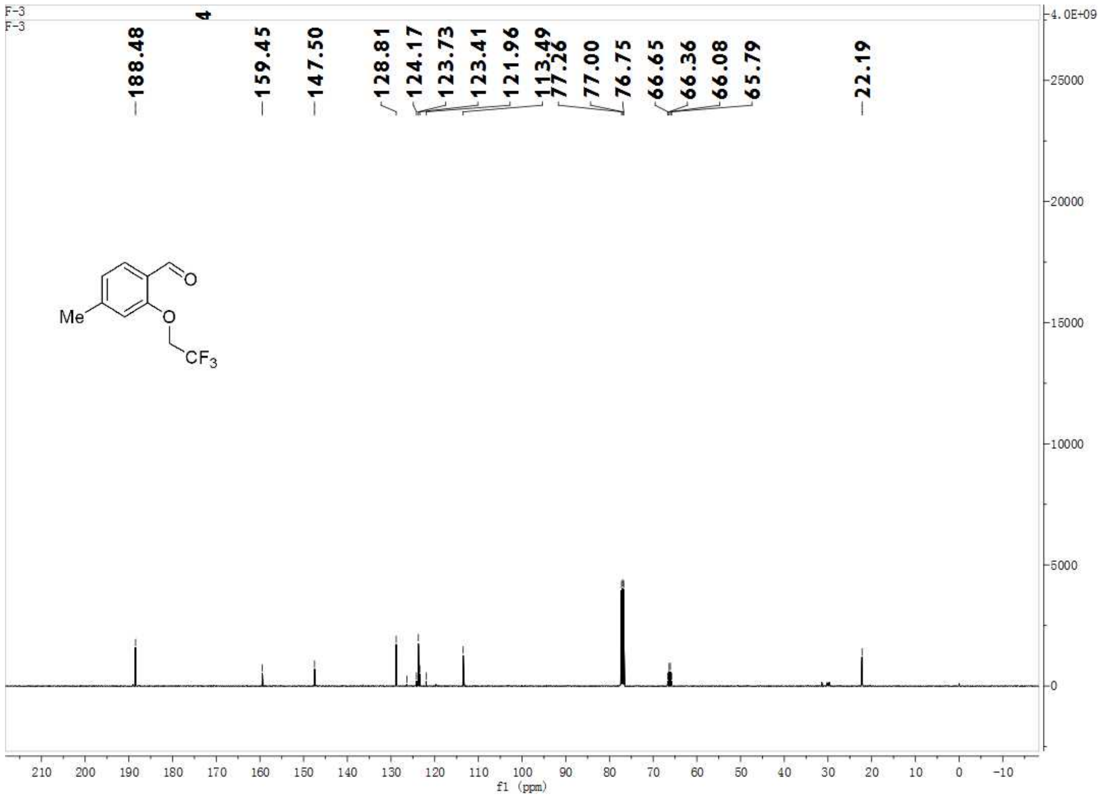

^{13}C NMR (125 MHz, $CDCl_3$) spectrum of compound **3b**

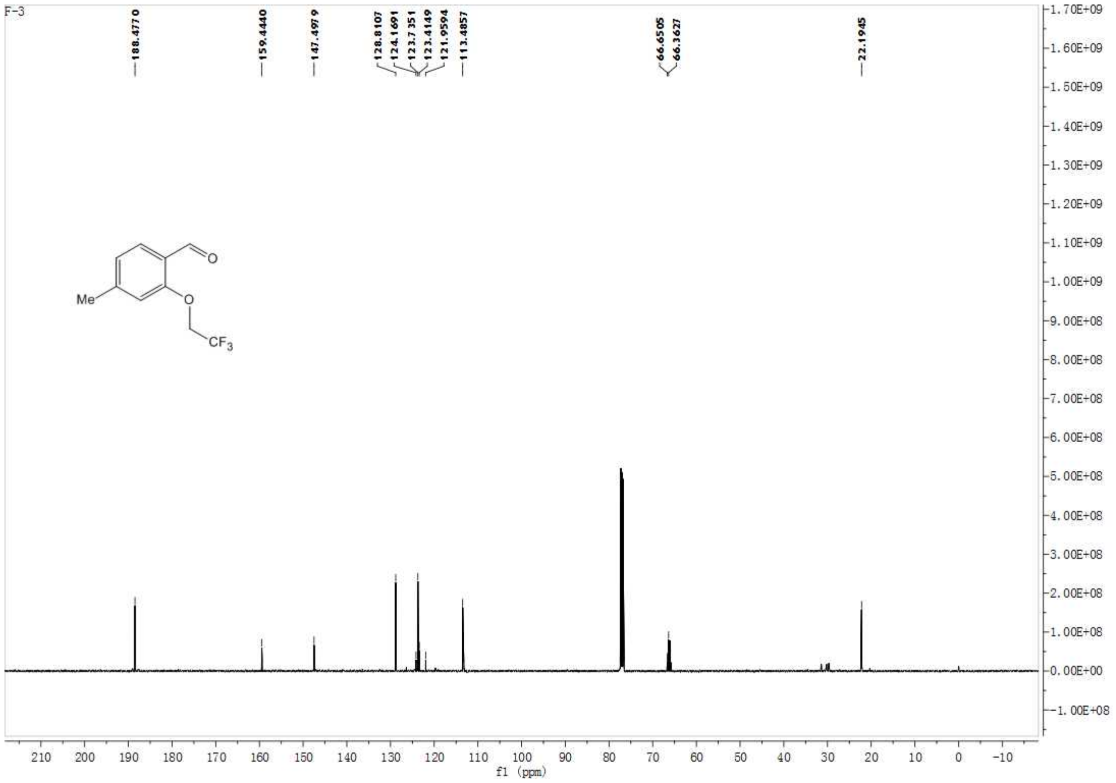

^{1}H NMR (500 MHz, $CDCl_3$) spectrum of compound **3c**

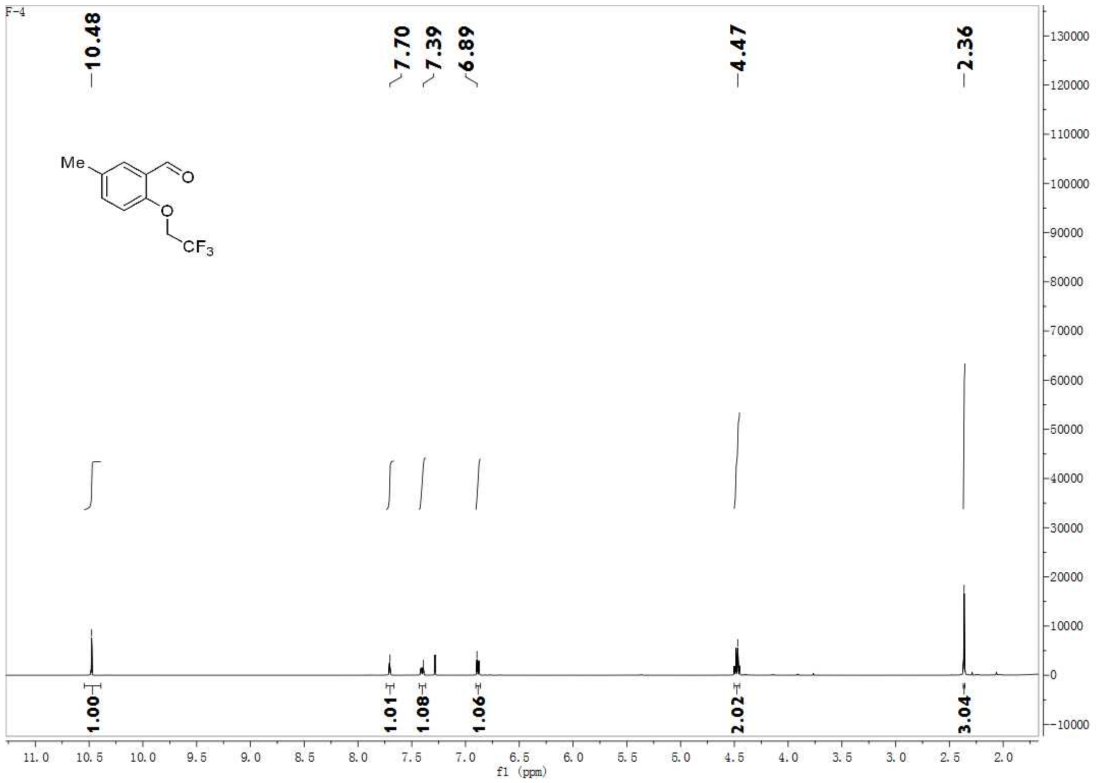

^{13}C NMR (125 MHz, $CDCl_3$) spectrum of compound **3c**

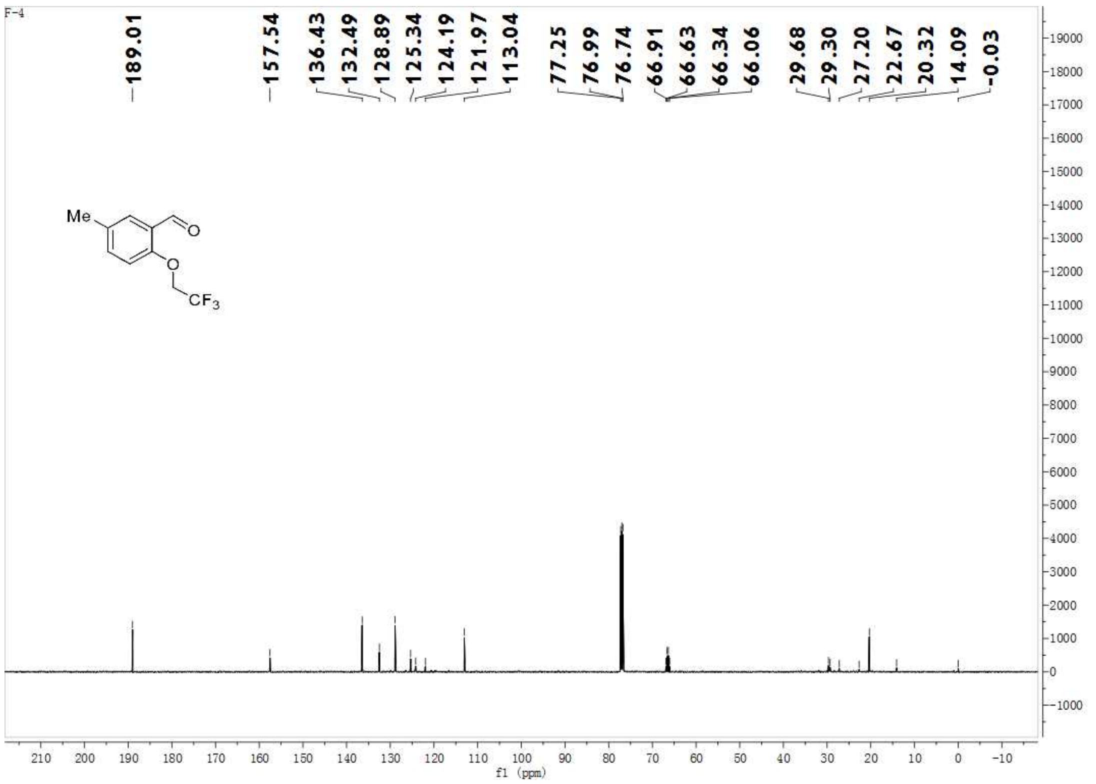

^{1}H NMR (500 MHz, $CDCl_3$) spectrum of compound **3d**

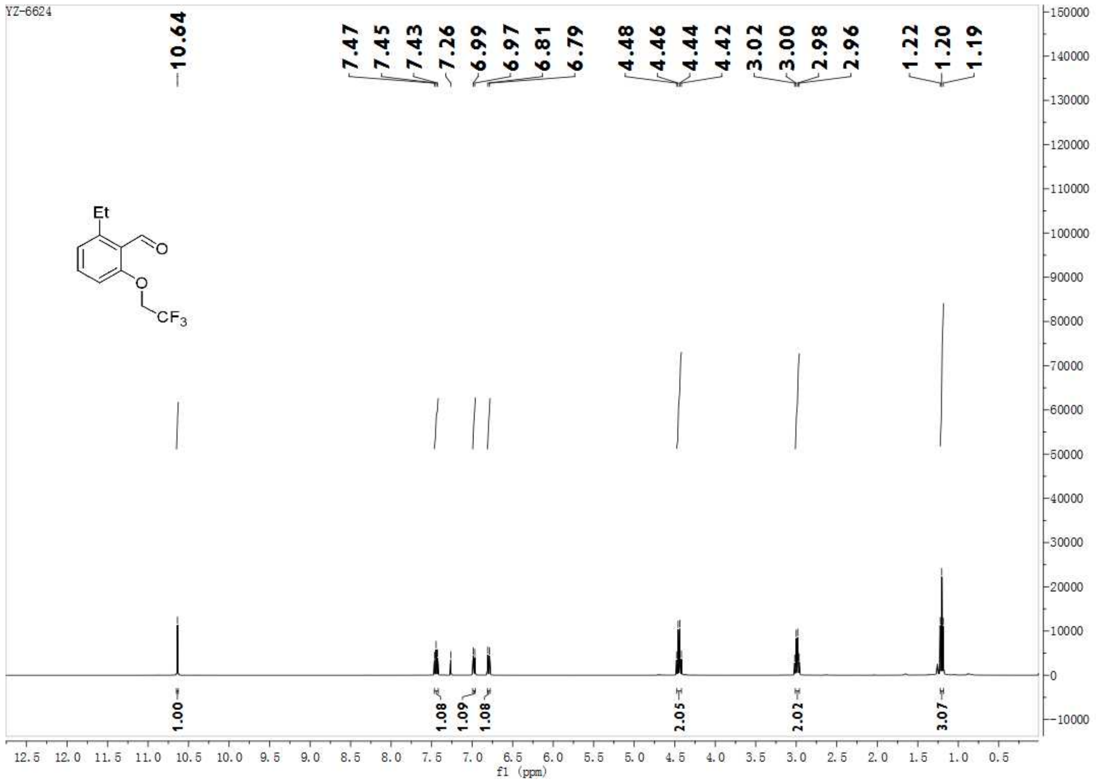

^{13}C NMR (500 MHz, $CDCl_3$) spectrum of compound **3d**

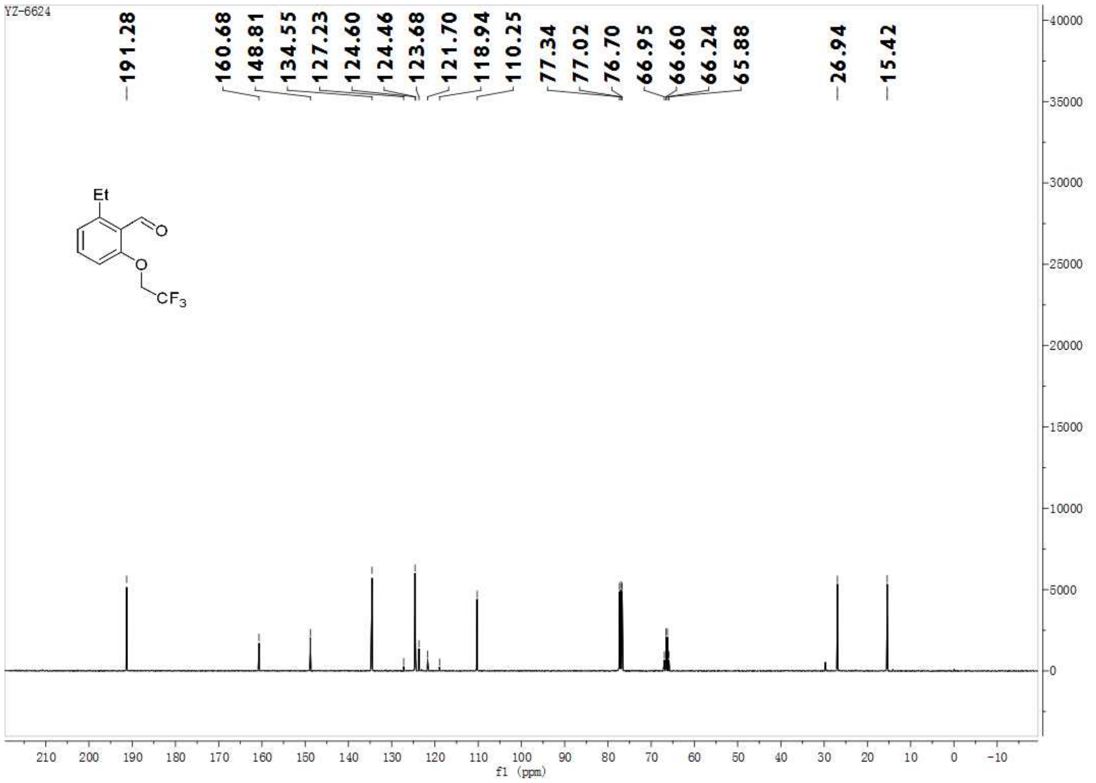

[1] H NMR (500 MHz, $CDCl_3$) spectrum of compound **3e**

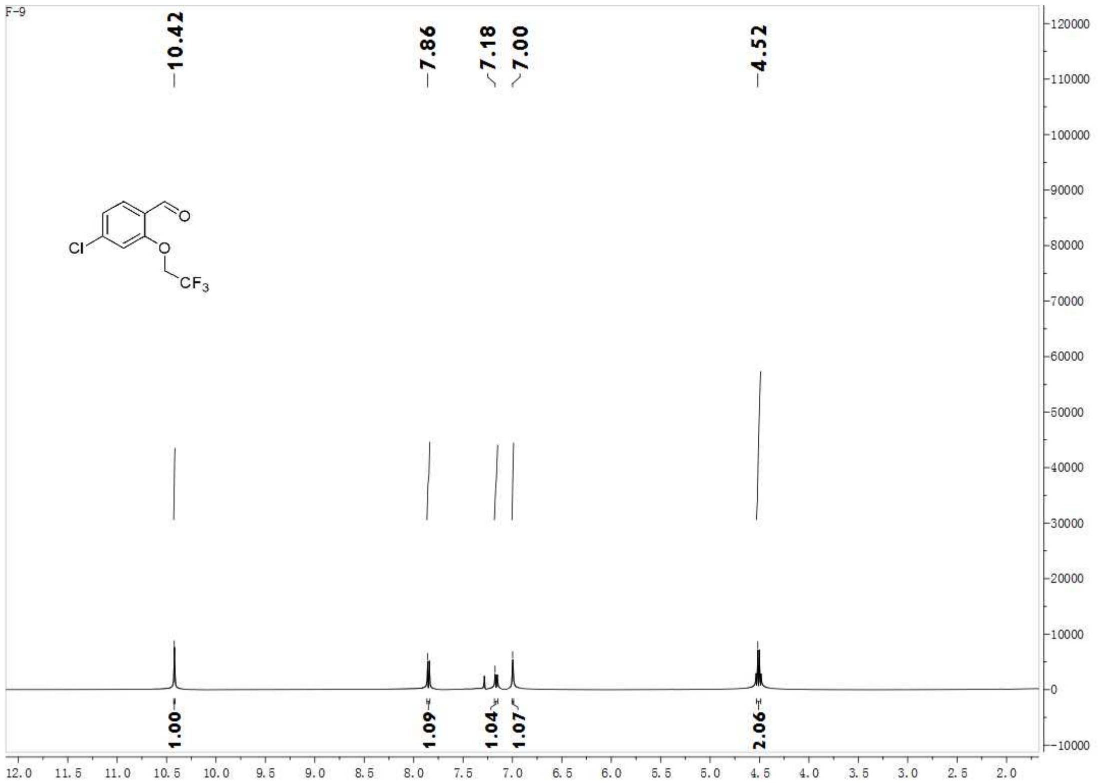

[13] C NMR (125 MHz, $CDCl_3$) spectrum of compound **3e**

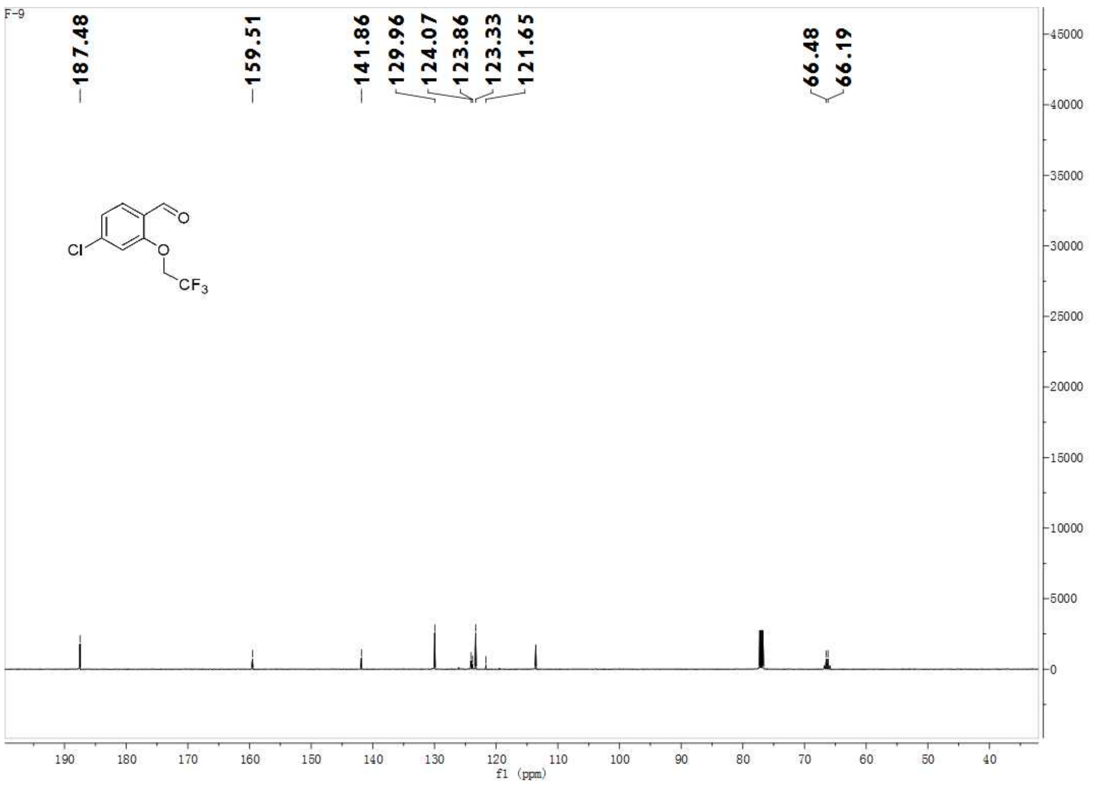

[1] H NMR (500 MHz, $CDCl_3$) spectrum of compound **3f**

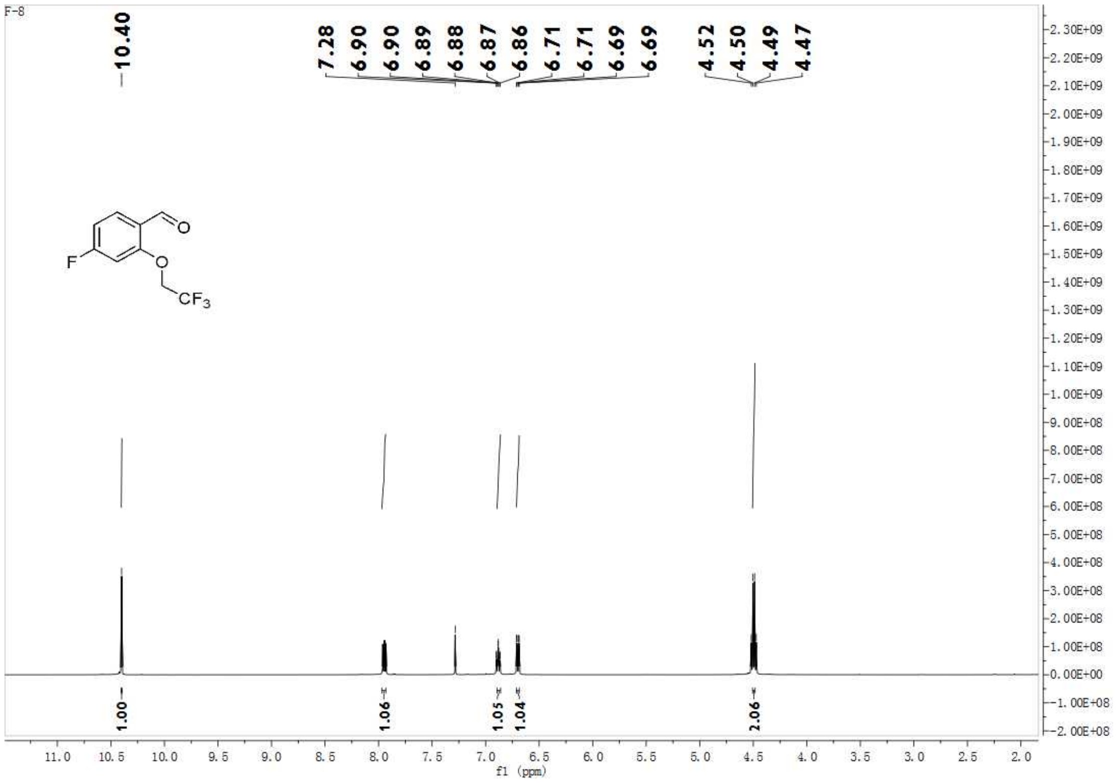

[13] C NMR (125 MHz, $CDCl_3$) spectrum of compound **3f**

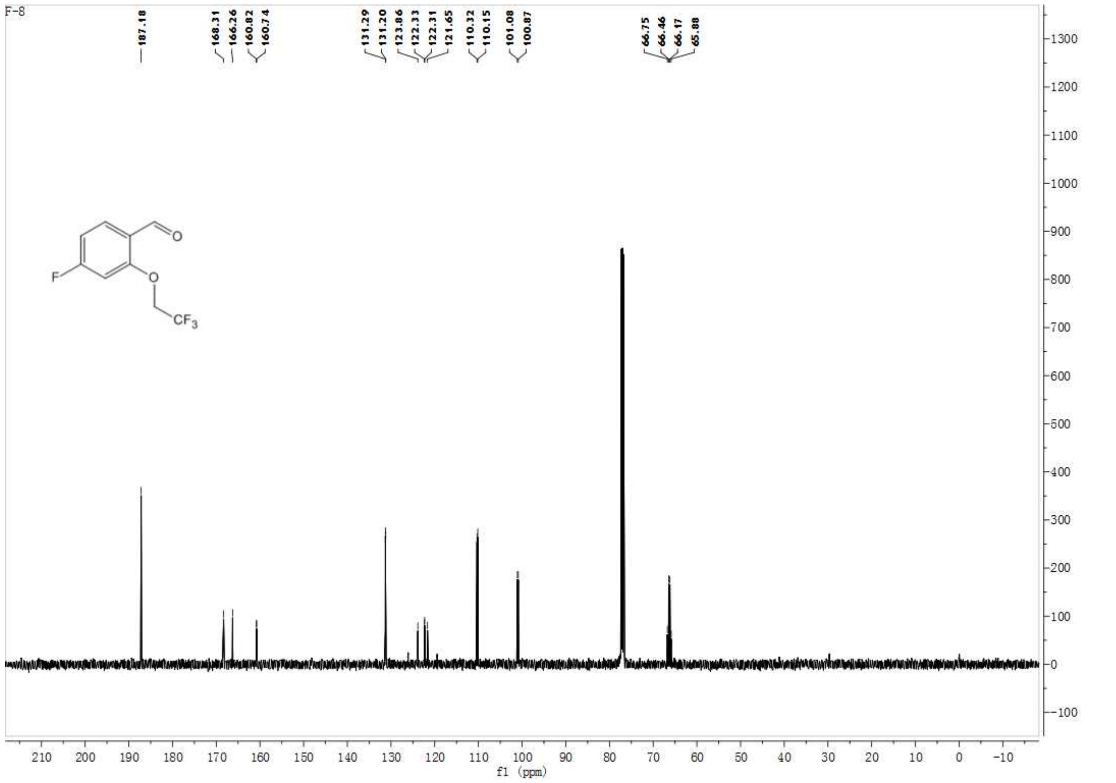

^{1}H NMR (500 MHz, $CDCl_3$) spectrum of compound **3g**

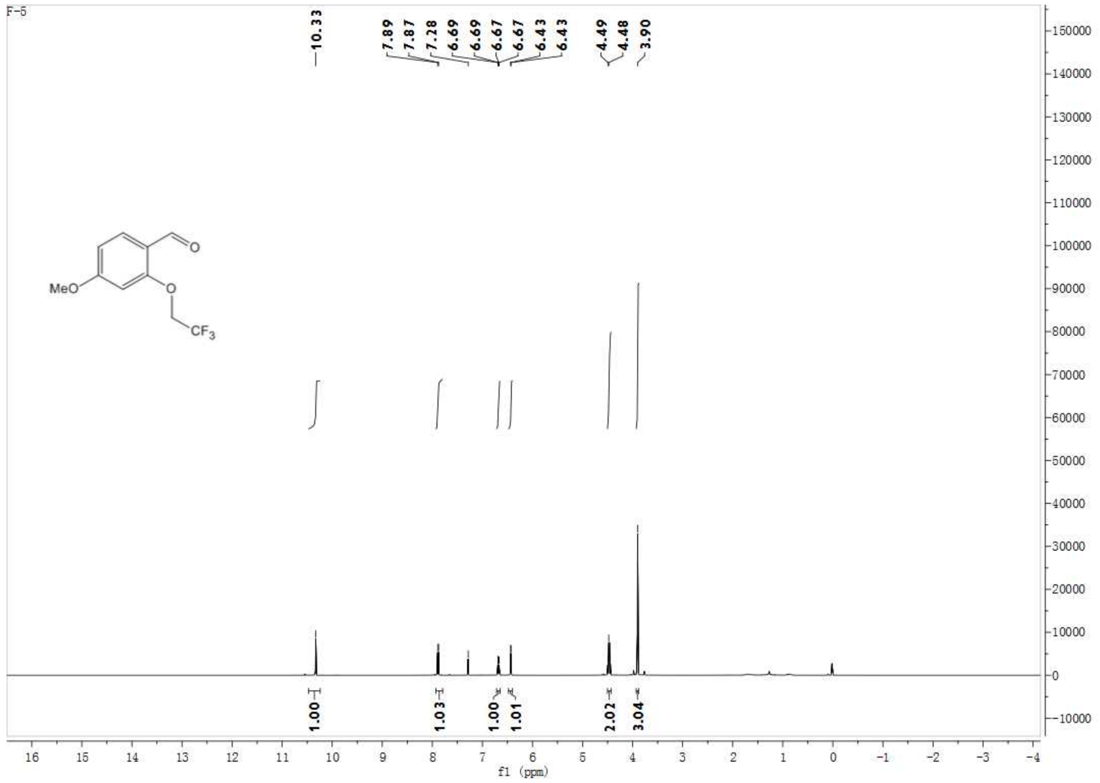

^{13}C NMR (125 MHz, $CDCl_3$) spectrum of compound **3g**

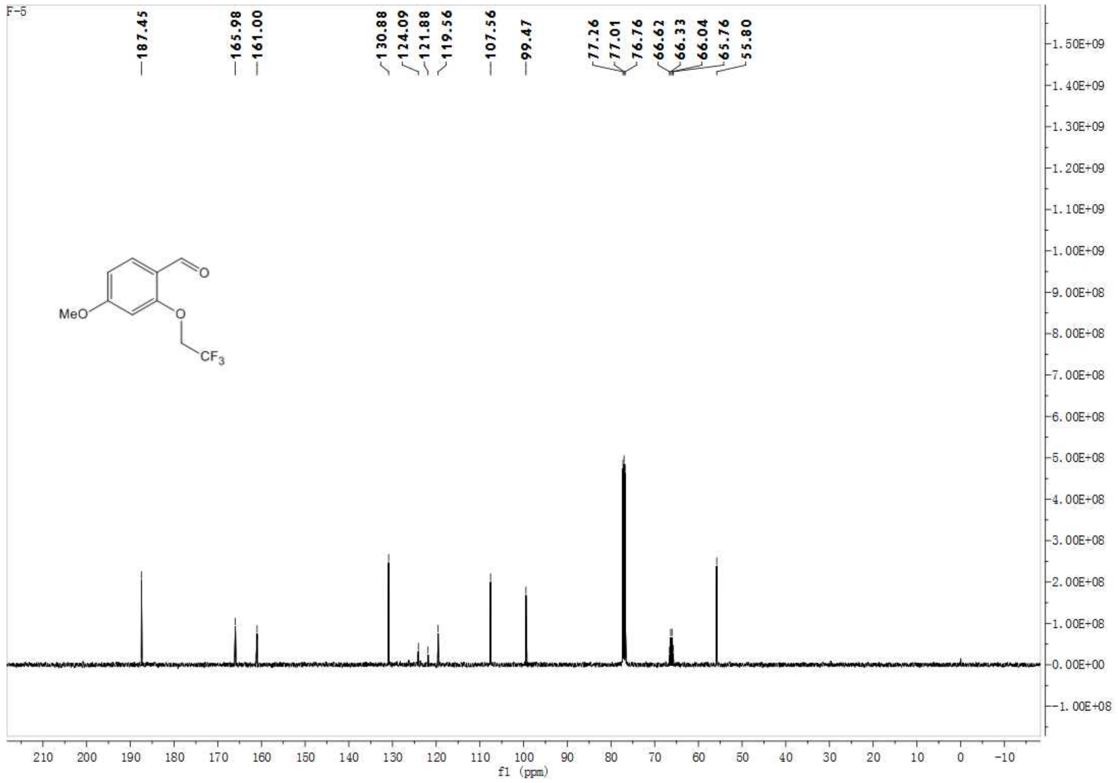

[1] H NMR (500 MHz, $CDCl_3$) spectrum of compound **3h**

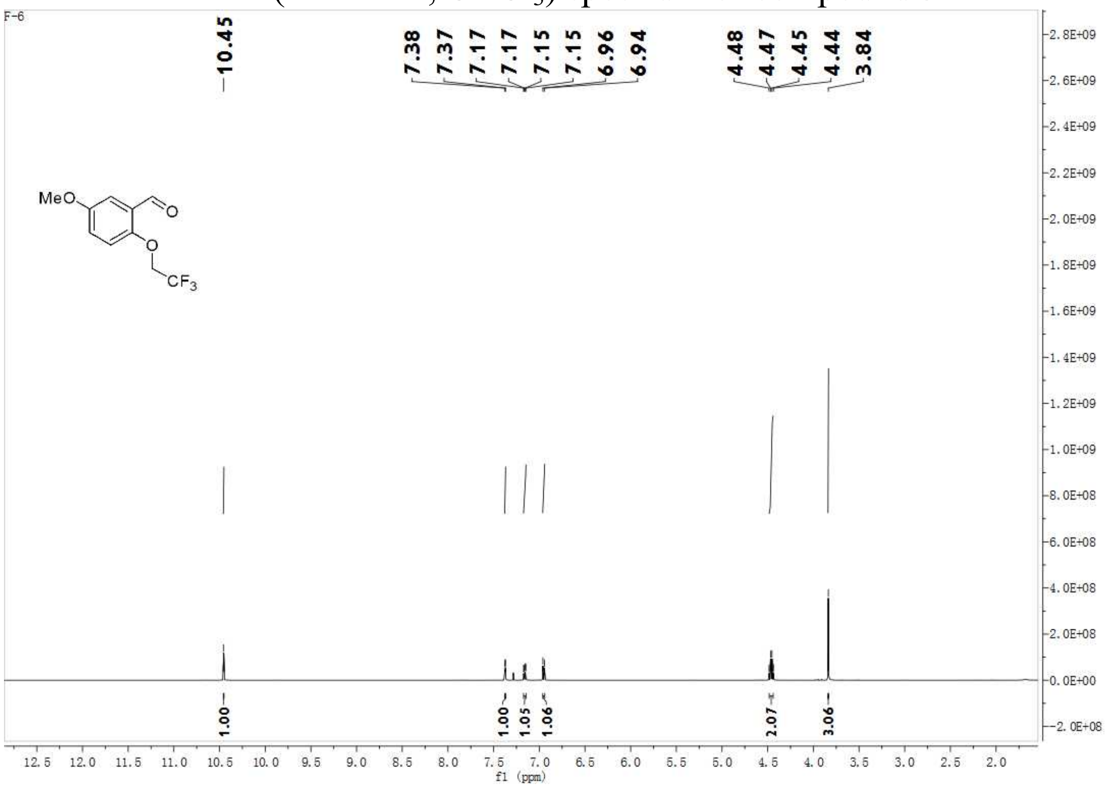

[13] C NMR (125 MHz, $CDCl_3$) spectrum of compound **3h**

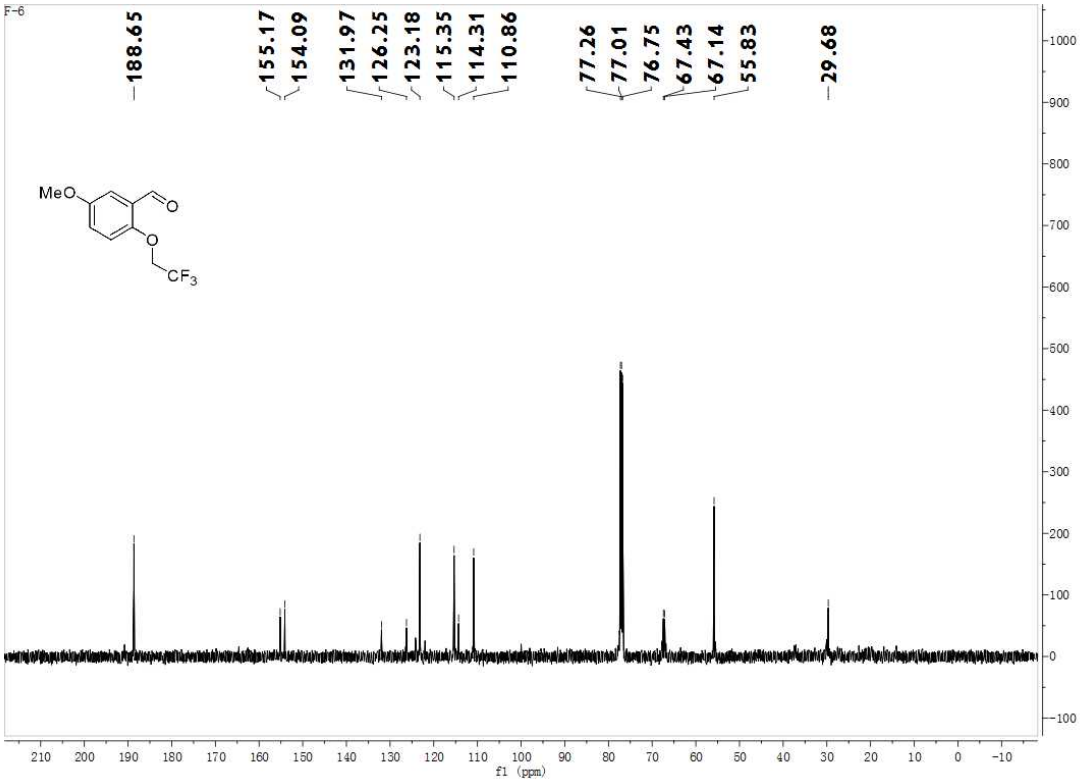

[1] H NMR (500 MHz, $CDCl_3$) spectrum of compound **3i**

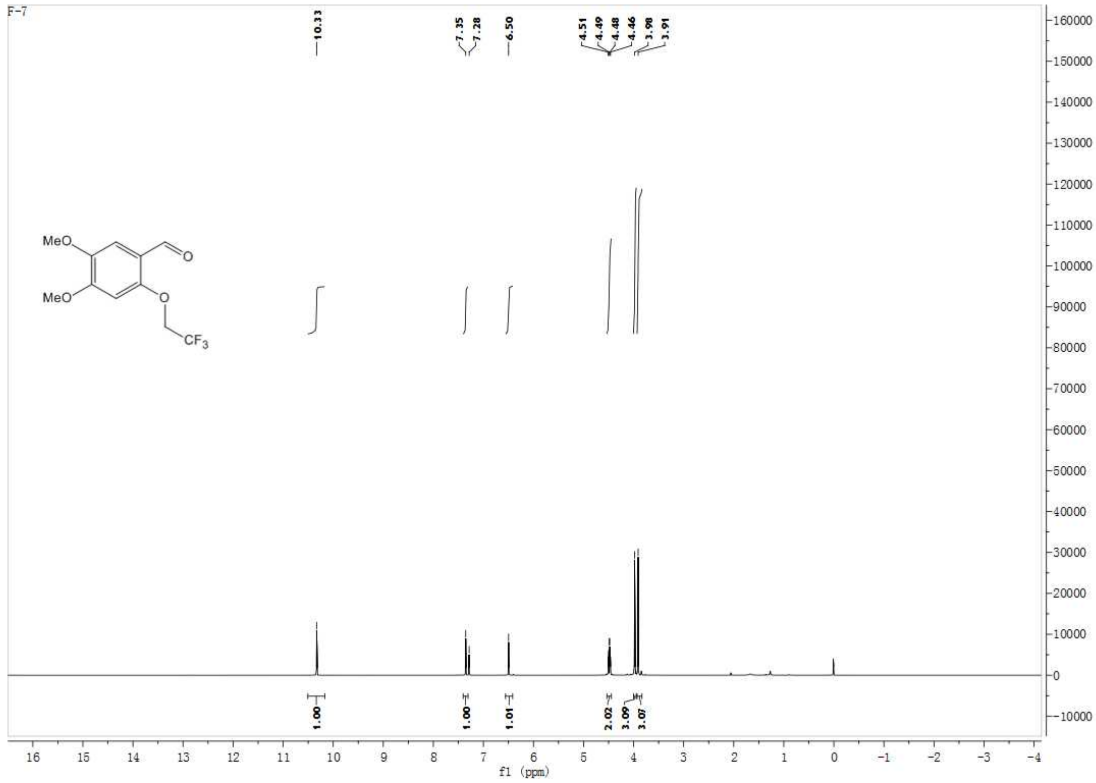

[13] C NMR (125 MHz, $CDCl_3$) spectrum of compound **3i**

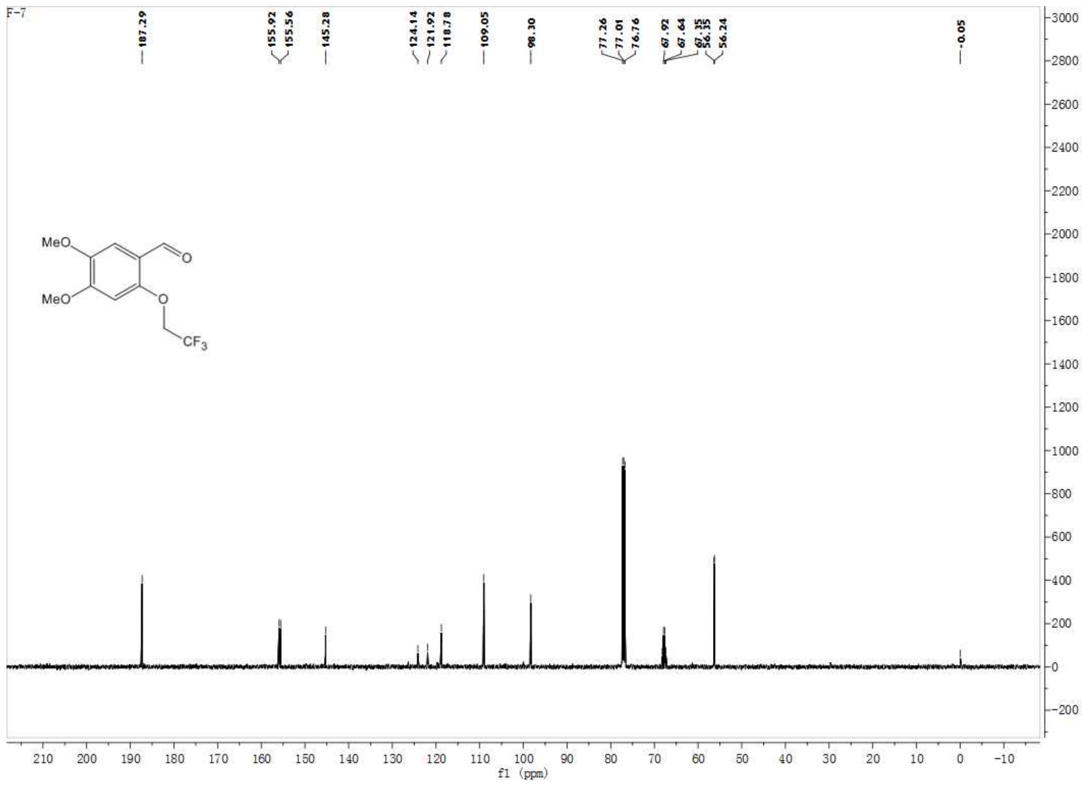

^{1}H NMR (500 MHz, $CDCl_3$) spectrum of compound **3l**

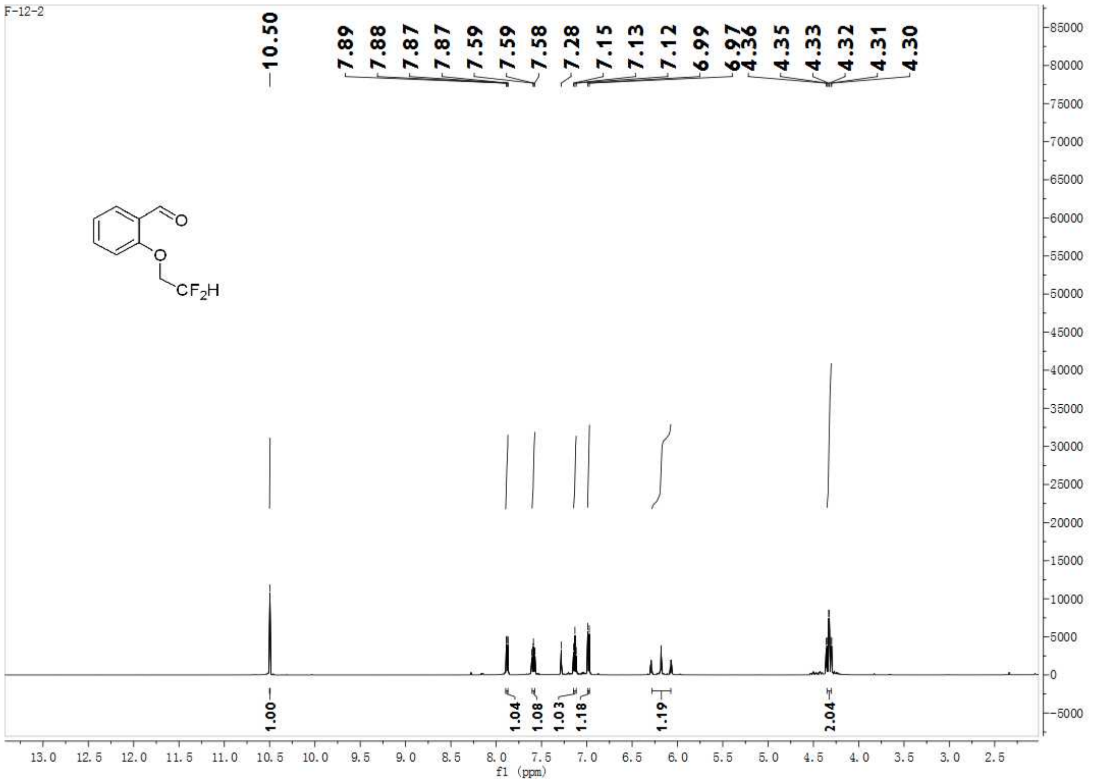

^{1}H NMR (500 MHz, $CDCl_3$) spectrum of compound **3m**

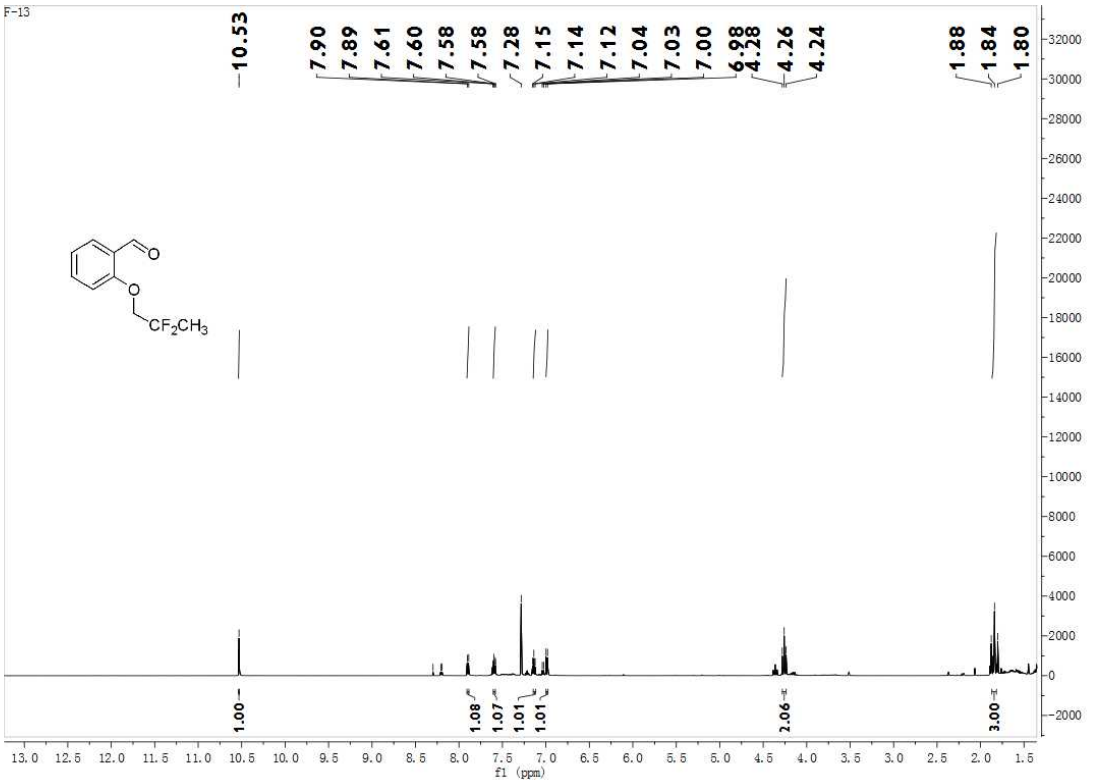

^{1}H NMR (500 MHz, $CDCl_3$) spectrum of compound **3n**

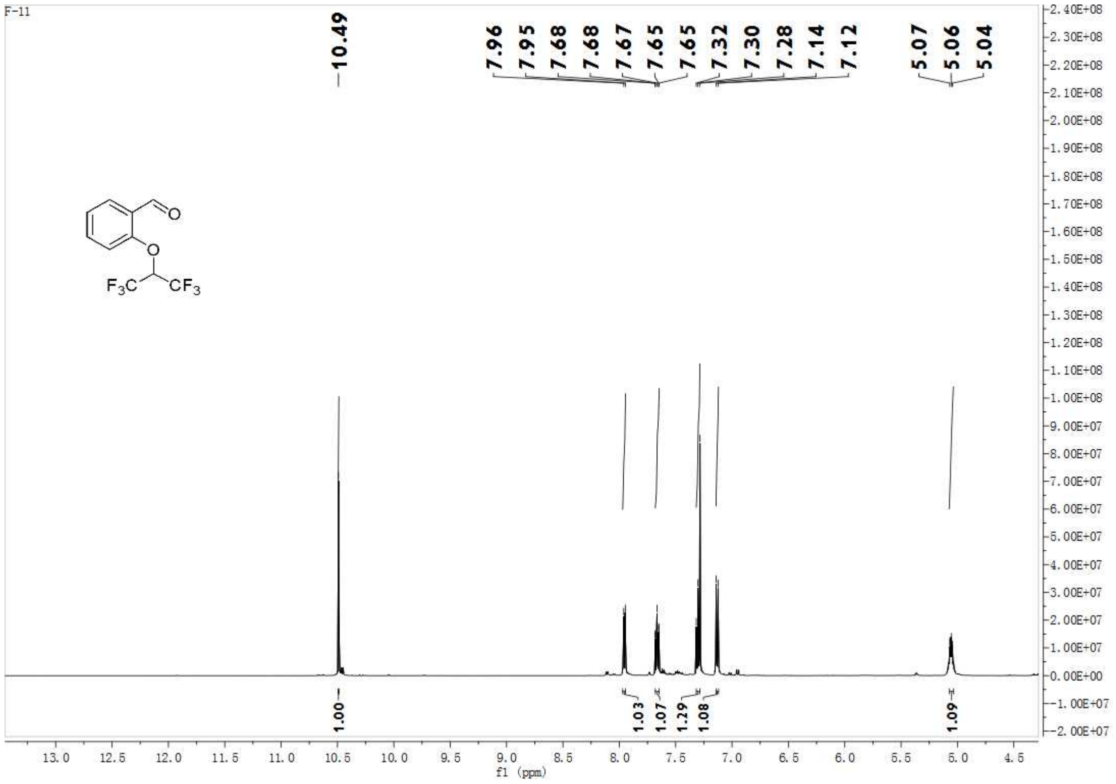

^{1}H NMR (500 MHz, $CDCl_3$) spectrum of compound **3o**

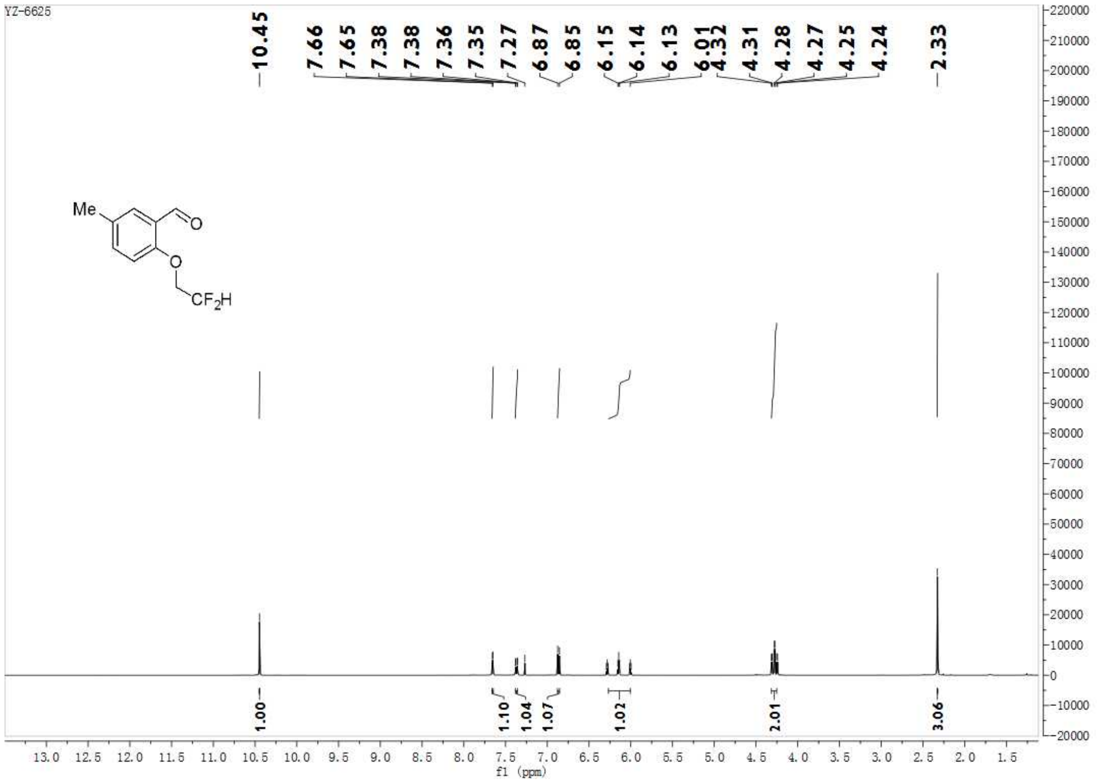

^{13}C NMR (500 MHz, $CDCl_3$) spectrum of compound **3o**

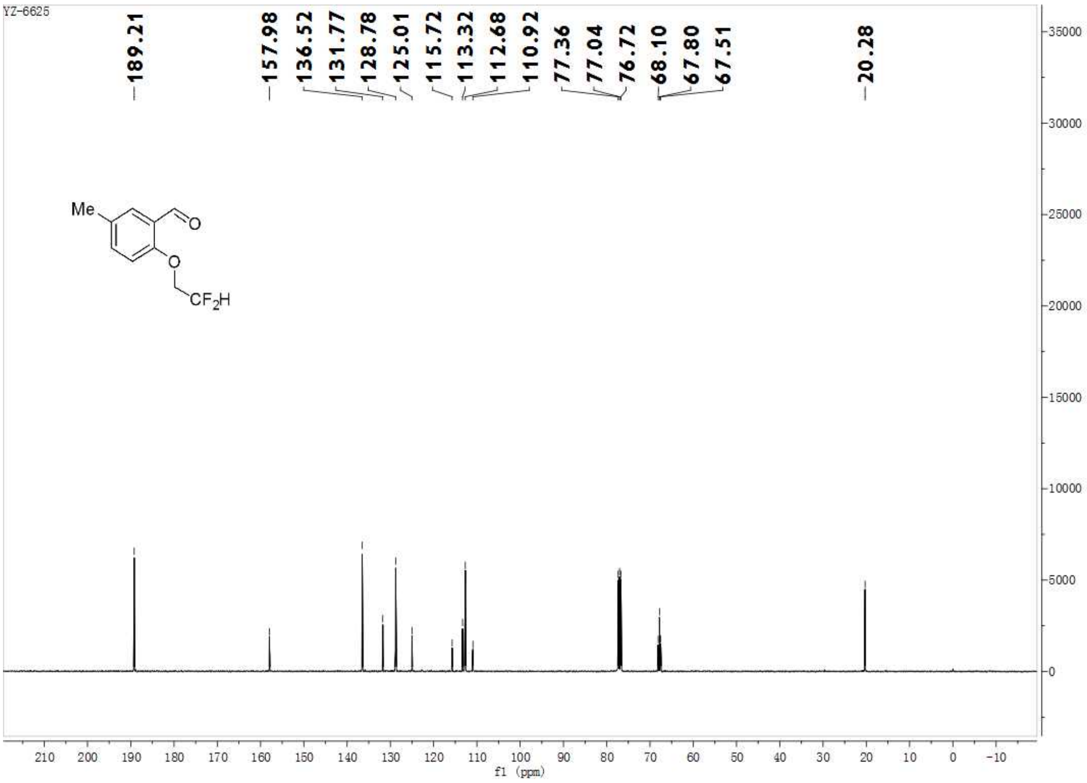

^{1}H NMR (500 MHz, $CDCl_3$) spectrum of compound **3p**

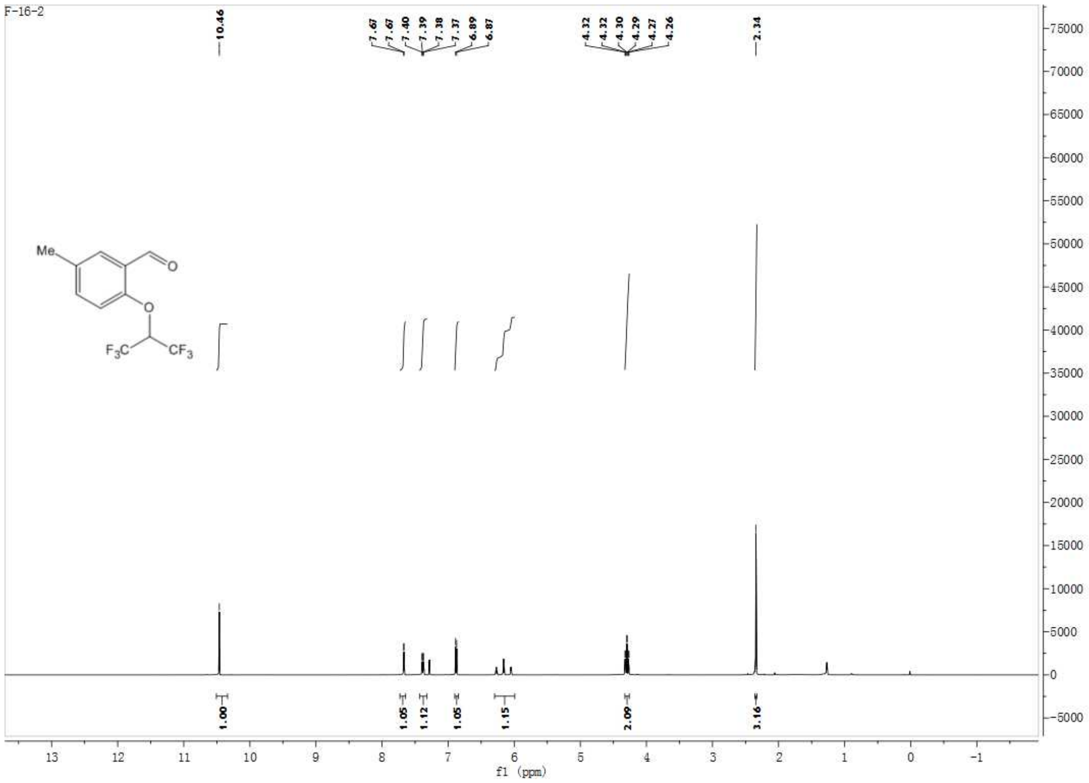

[1] H NMR (500 MHz, $CDCl_3$) spectrum of compound **3q**

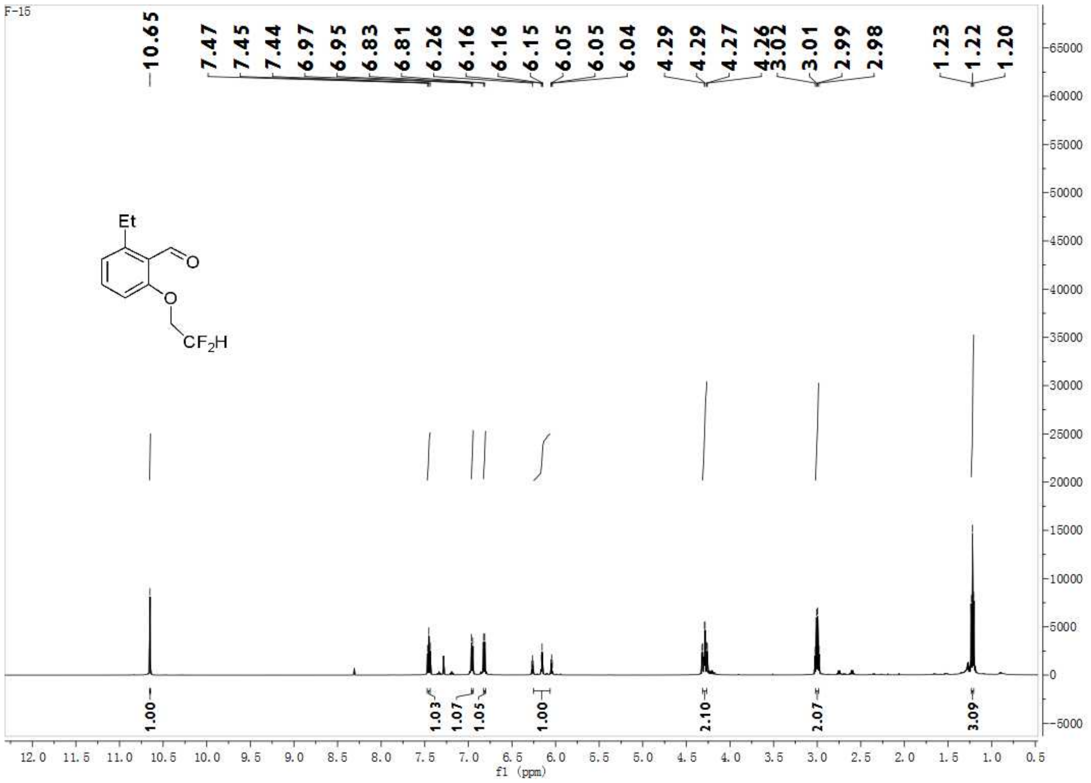

[13] C NMR (500 MHz, $CDCl_3$) spectrum of compound **3q**

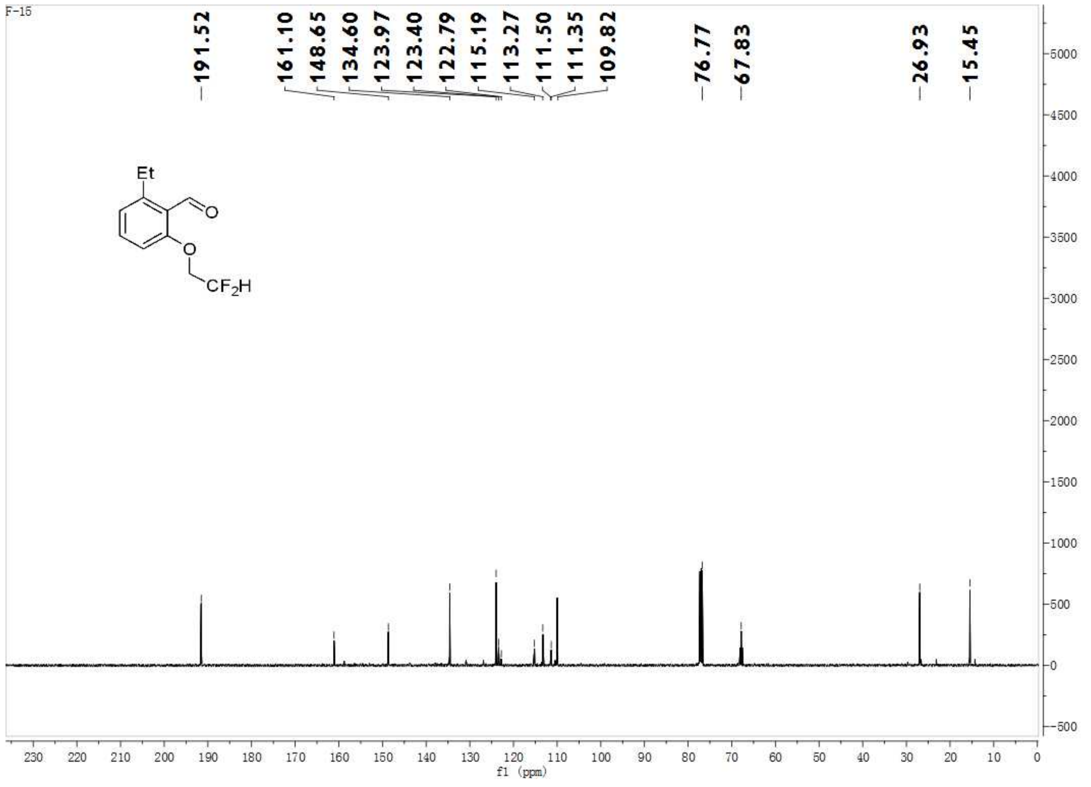

[1] H NMR (500 MHz, $CDCl_3$) spectrum of compound **3r**

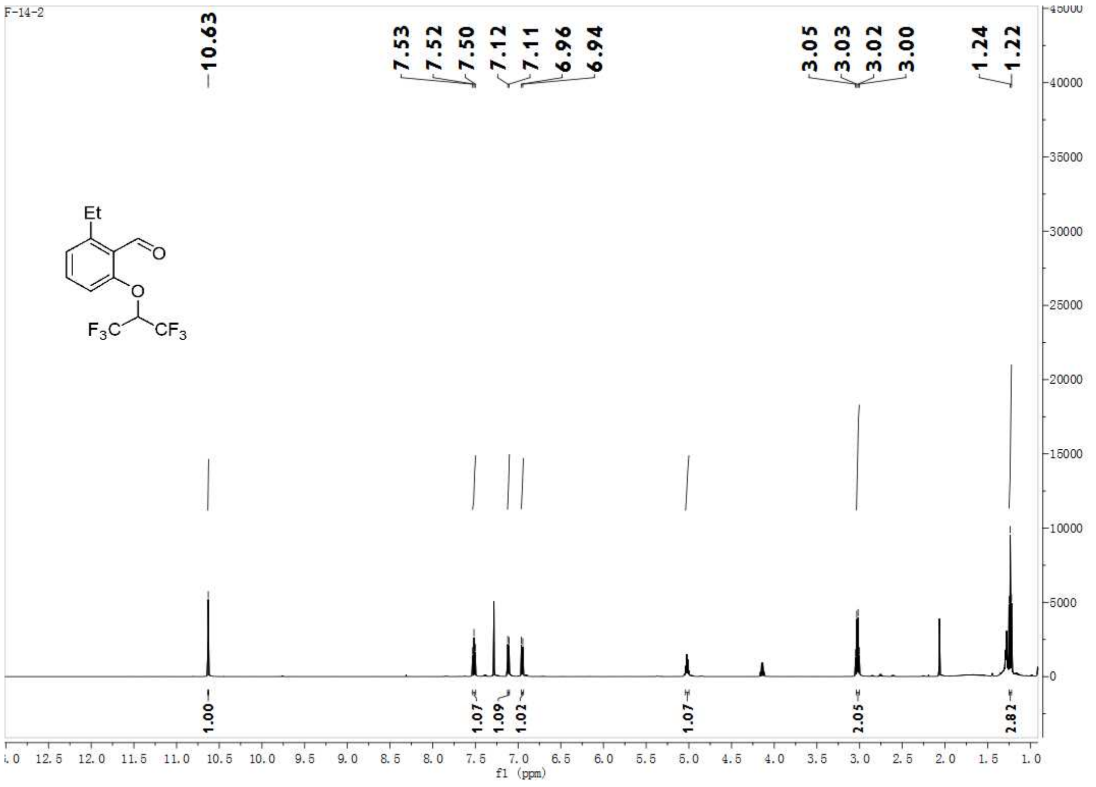

[1] H NMR (500 MHz, $CDCl_3$) spectrum of compound **3s**

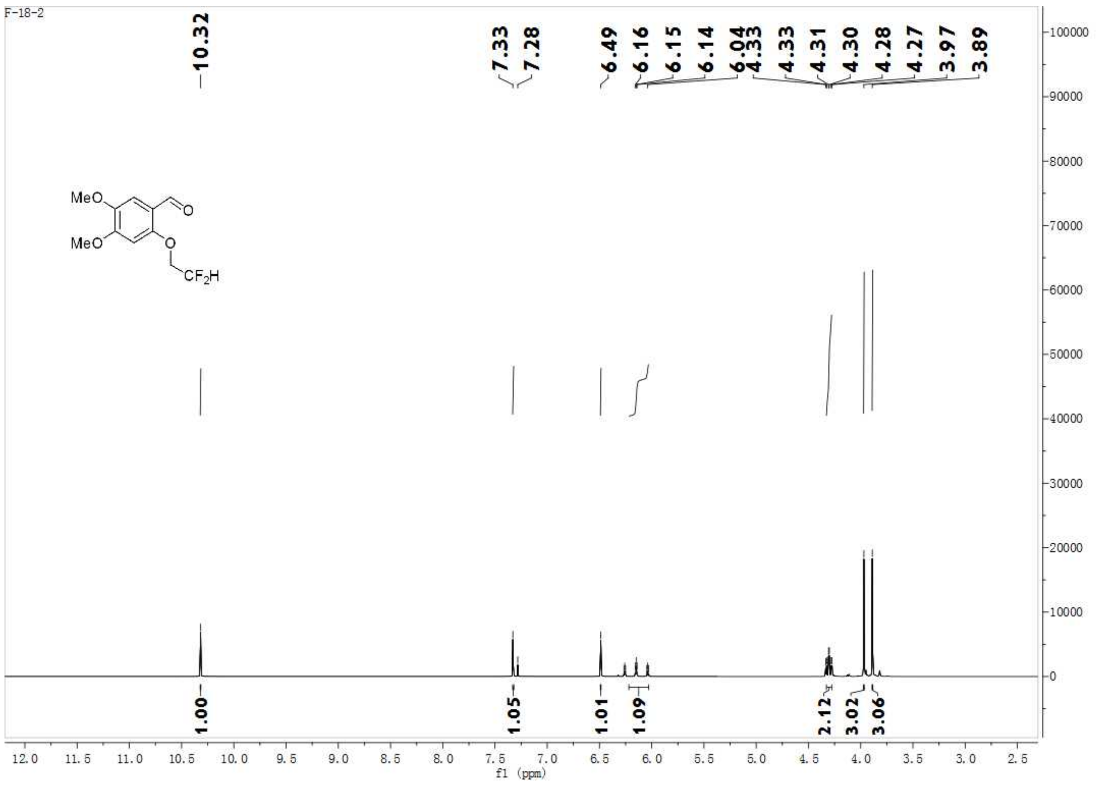

Supplement: RA-012-D2RA00241H-s001 [file RA-012-D2RA00241H-s001.pdf]
